# Cytoplasmic HMGB1 promotes and interacts with BECN1 through ZNF460 to induce autophagy and accelerate radioresistance in colorectal cancer cells

**DOI:** 10.3389/fimmu.2025.1642915

**Published:** 2025-10-14

**Authors:** Yuhui Han, Xiuxin Liu, Wenjiong Sheng, Ruixue Kuang, Yan Zhang, Xinyu Jia, A. M. Abd El-Aty, Tao Hu, Bin Wang, Yanchao Ma

**Affiliations:** ^1^ Department of Immunology, Binzhou Medical University, Shandong, China; ^2^ Department of Radiation Therapy, Yantaishan Hospital, Affiliated Hospital of Binzhou Medical University, Shandong, China; ^3^ Department of Clinical Laboratory, Yantaishan Hospital, Affiliated Hospital of Binzhou Medical University, Shandong, China; ^4^ Department of Pharmacology, Faculty of Veterinary Medicine, Cairo University, Giza, Egypt; ^5^ Department of Medical Pharmacology, Medical Faculty, Ataturk University, Erzurum, Türkiye

**Keywords:** colorectal cancer, radioresistance, HMGB1, BECN1, autophagy, ZNF460

## Abstract

Radioresistance results in relapse and treatment failure in locally advanced colorectal cancer (CRC) patients. HMGB1 is reportedly associated with radioresistance in esophageal squamous cell carcinoma and breast cancer. However, its role in the response of CRC to radiotherapy has not been fully elucidated. Thus, we explored the role and underlying mechanism of HMGB1 in CRC radioresistance. The total amount of HMGB1 and its translocation from the nucleus to the cytoplasm increased after irradiation. Functional studies revealed that HMGB1 enhanced the proliferation and autophagy of CRC cells after irradiation. Mechanistically, HMGB1 can regulate the transcription factor ZNF460, which combines with the BECN1 promoter to promote the release of BECN1 into the cytoplasm after irradiation. Moreover, HMGB1 directly interacts with BECN1 in the cytoplasm, thereby resulting in CRC radioresistance. Finally, the protein expression levels of BECN1, which was positively correlated with HMGB1, were significantly increased in human CRC tissues and associated with TNM stage and poor prognosis in patients with CRC. Our findings revealed that HMGB1 plays a vital role in CRC radioresistance by regulating autophagy through binding with BECN1. Given the efficacy of HMGB1 modulation in CRC suppression and radioresistance, HMGB1 has emerged as a potential therapeutic molecule for CRC treatment.

## Introduction

Colorectal cancer (CRC) is the third most common malignancy and the second leading cause of cancer-related mortality worldwide (1). Previous studies have shown that neoadjuvant chemoradiotherapy can facilitate complete tumor resection for locally advanced CRC, depending on tumor downstaging and even clinical and pathological complete remission (2). However, not all patients with CRC respond positively to radiotherapy. Approximately 50–60% of patients with locally advanced rectal cancer achieve a partial response, whereas 15–20% achieve a pathological complete response (3). Moreover, approximately 35% of patients with CRC experience recurrence and metastasis within 5 years after surgery and chemoradiotherapy, which results in mortality in patients with distant relapse (4). In brief, tumor cells evade the biological effects caused by radiotherapy, leading to radioresistance and ultimately to the failure of radiotherapy. Therefore, biomarkers to evaluate the prognosis of CRC patients receiving radiotherapy are urgently needed, and elucidating the molecular mechanism underlying CRC radioresistance to prolong the survival time and accelerate the quality of life of patients with CRC is urgently needed.

High mobility group box 1 (HMGB1) is a nonhistone DNA-binding protein that is widely expressed in the nucleus, cytoplasm, and extracellular region and has diverse functions, such as influencing cell migration, cell differentiation, inflammation, and tumor progression (5). HMGB1 expression is ubiquitously upregulated in various tumors, including breast cancer, lung cancer, and hepatocellular carcinoma (6–8). In addition, HMGB1 can translocate into the cytoplasm for release under exposure to various stressors, including chemotherapy and radiotherapy (6). Our previous study revealed that HMGB1 expression is abnormal in CRC, which is related to the DNA repair response (9). Moreover, as an acknowledged inhibitor of HMGB1, glycyrrhizin exerts anticancer effects on CRC cells by inducing apoptosis and autophagy and suppressing cell migration and invasion (10). Multiple studies have shown that HMGB1 is involved in the chemoresistance of CRC (11, 12). Nevertheless, the functional roles of HMGB1 associated with radioresistance in CRC have yet to be investigated.

Autophagy plays a vital role in tumor radioresistance (13). Autophagy can reduce the radiosensitivity of tumor cells by affecting DNA repair and reactive oxygen species clearance (2). Additionally, HMGB1 promotes beclin 1 (BECN1)-dependent autophagy, which subsequently promotes radioresistance in oral squamous or esophageal squamous cell carcinoma (ESCC) (14, 15). In this study, we aimed to explore the biological functions of HMGB1 in the response to radiotherapy both *in vitro* and *in vivo*, the internal functional mechanisms, and the potential relationship of this protein with BECN1-related autophagy.

## Materials and methods

### Cell lines and culture

The normal colonic epithelial cells (NCM460, RRID: CVCL_0460) and CRC cells (HCT116, RRID: CVCL_0291, HT29, RRID: CVCL_0320, DLD-1, RRID: CVCL_0248, SW480, RRID: CVCL_0546, SW620, RRID: CVCL_0547) were purchased from Servicebio Technology Co., Ltd. (Wuhan, China) with cell line authentication. All the cells were cultured in DMEM (CellMax, Beijing, China) supplemented with 10% fetal bovine serum (FBS; CellMax, Beijing, China), 100 U/mL penicillin, and 100 mg/mL streptomycin (Solarbio, Beijing, China) in a humidified atmosphere containing 5% CO_2_ at 37°C. All the cell lines were periodically tested to confirm that they remained mycoplasma free.

### Irradiation

The radiation dose was as previously described (16). The HCT116 and HT29 cells were cultivated and exposed to a radiation dose of 4 Gy for cell proliferation, apoptosis and cell cycle analyses via X-ray irradiation (Precision X-ray. Inc., Glendale, CA, USA) with a dose rate of 1.5 Gy/min. Similarly, subcutaneous tumor-bearing mice were subjected to local radiotherapy at a dose of 10 Gy.

### Cell transfection and lentiviral infection

Lentiviral vectors carrying human HMGB1 shRNA were generated by GeneChem Co., Ltd. (Shanghai, China). The empty vector control lentivirus (sh-NC) was used as the control. For lentivirus infection, HCT116 and HT29 cells at 30% confluence in 6-well plates were transduced with lentiviral particles at an MOI of 10. After 48 h of lentivirus infection, the infection efficiency was validated by counting GFP-expressing cells under a fluorescence microscope at 72 h after infection. si-BECN1, si-ZNF460 (RIBOBIO, Guangzhou, China) and the negative control were transfected into cells via Lipo2000 (Vazyme, Nanjing, China) transfection reagent according to the manufacturer’s instructions. The transfection efficiency was determined via RT–qPCR and western blotting.

### RNA isolation and RT–qPCR

Total RNA was extracted from cultured cells via RNAiso reagent (Vazyme, Nanjing, China) according to the manufacturer’s instructions. A total of 1 μg of RNA was reverse-transcribed via a HiScript II One Step RT–PCR Kit (Vazyme), and the acquired cDNA was analyzed in triplicate via real-time PCR on an ABI 7500 Real-Time PCR system (Applied Biosystems, Waltham, MA, USA) via ChamQ SYBR qPCR Master Mix (Vazyme). The PCR conditions were as follows: 95 °C for 30 s, followed by 40 cycles of amplification for 3 s at 95 °C and 30 s at 60 °C. Individual gene expression was normalized to that of GAPDH mRNA. The primers used for amplification are listed in **Supplementary Table S1**.

### Western blotting

HCT116 or HT29 cells in 6-well plates were lysed with RIPA lysis buffer (Solarbio) containing a protease inhibitor cocktail (Solarbio) according to the manufacturer’s instructions. The protein concentrations were quantified via a Pierce BCA protein assay kit (Vazyme). Equal amounts of protein were separated by 12% SDS–PAGE and transferred to PVDF membranes (Merck Millipore, Darmstadt, Germany). The membranes were incubated with appropriate diluted primary antibodies overnight at 4 °C. The second day, the PVDF membranes were washed four times with TBST before they were incubated with horseradish peroxidase-conjugated secondary antibodies (Proteintech) for 1 h at room temperature. The films were subsequently developed with Clarity Western ECL substrate (Elabscience, Hubei, China) and visualized with a ChemiDoc™ MP imaging system (Tacan, Shanghai China). Information about the antibodies is presented in **Supplementary Table S2**.

### CCK-8 assay

The cells (5 × 10^3^ cells/well) were seeded into 96-well plates and exposed to 4 Gy X-ray radiation for 24 h. The cells were subsequently stained with 10 μL of sterile CCK-8 solution (Dojindo Laboratory, Mashikimachi, Japan) for 4 h at 37°C. Finally, the OD was measured at 450 nm to determine cell viability.

### Formation of cell colonies

The cells were seeded in 12-well plates at different densities (500, 500, 1000, 1000, and 1000 cells) and exposed to 0, 2, 4, 6, or 8 Gy of radiation. After 2 weeks, the colonies were fixed in methanol for 30 min and then stained with 1% crystal violet (Solarbio) for 30 min. Finally, cell colonies with more than 50 cells were counted. The radiation sensitivity-enhancement ratio (SER) was measured via a “single-hit multitarget” model.

### Flow cytometry

The cells were harvested 24 h after 4 Gy irradiation and washed with cold PBS, and the cell cycle distribution and apoptosis rate were analyzed via flow cytometry (Beckman Coulter, CA, USA). Briefly, CRC cells were plated into 6-well plates at 2 × 10^5^ cells per well and then exposed to 0- or 4-Gy X-ray radiation for 24 h. To analyze the cell cycle distribution, the cells were washed twice with PBS and fixed in 70% ethanol containing 0.5% FBS. The samples were treated with cell cycle reagent (Solarbio) and analyzed via flow cytometry via ModFit LT 5.0. For apoptosis analysis, the cells were stained with Annexin V-PE and 7-AAD (Elabscience) according to the manufacturer’s instructions.

### Xenograft mouse model

Female BALB/c nude mice (6–8 weeks, 18–20 g) were purchased from Shanghai Model Organisms Center, Inc., and maintained under specific pathogen-free conditions. All animal experiments were approved by the Institutional Ethics Committee of Binzhou Medical University. All the mice were synchronized with a 12 h light/dark cycle in an autonomous chronobiological animal facility (Yantai, Shandong, China), with lights on from 6 am (Zeitgeber time 0) to 6 pm (Zeitgeber time 12) for 1 week.

To generate tumors, mice were injected subcutaneously with 5 × 10^6^ sh-NC or sh-HMGB1-HT29 cells suspended in 100 μl of sterile PBS in the flanks of each mouse on day 0. The mice were randomly assigned to receive 10 Gy irradiation or Tat-BECN1 (15 mg/kg, Selleck, Shanghai, China) injected intraperitoneally (17). Tumor growth was monitored by measuring the length (a) and width (b) of the tumor every 2 days, and the tumor volume was calculated via the formula V = ab^2^/2. After 24 days, the mice were euthanized by cervical dislocation, and the tumors were harvested, weighed, and processed for further analysis.

### TUNEL assay

For the apoptosis assay, xenograft tumor tissues from BALB/c nude mice were examined via a one-step TUNEL *In Situ* Apoptosis Kit (Elabscience) according to the manufacturer’s instructions. Briefly, sections from paraffin-embedded xenograft tumor tissues were dewaxed, rehydrated, and then incubated with a TUNEL reaction mixture at 37°C for 1 h in a chamber with a humidified atmosphere. The nuclei were stained with DAPI, and the numbers of TUNEL-positive cells and total cells were analyzed via a confocal microscope (Zeiss, Oberkochen, Germany).

### Coimmunoprecipitation

The Co-IP assay was performed via a Co-IP kit (Absin, Shanghai, China; abs955) according to the manufacturer’s instructions. CRC cells in 6-well plates were washed once with PBS and lysed in lysis buffer on ice for 30 min. Subsequently, the soluble cell lysate fractions were collected and centrifuged at 13,000 × g for 10 min at 4°C. One part of the cell lysate served as the positive control, and the other part of the protein fraction was soaked with a mouse anti-HMGB1 antibody and a mouse IgG antibody overnight at 4°C, followed by gentle mixing with protein A + G agarose beads at 4°C for 3 h. The beads were rinsed five times with wash buffer. Finally, BECN1 antibodies were tested via western blotting.

### Proximity ligation assay

The PLA was conducted via the Duolink^®^ *in situ* proximity ligation assay (#DUO92101) according to the manufacturer’s protocol (Sigma–Aldrich, New Jersey, USA). Briefly, the cells were fixed with 4% paraformaldehyde and permeabilized with 1% Triton X-100. The cells were subsequently incubated with blocking solution for 60 min and primary antibodies overnight at 4°C. The next day, the cells were incubated with secondary antibodies conjugated with oligonucleotides (anti-rabbit PLUS probe and anti-mouse MINUS probe) at 37°C for 60 min. Then, the cells were incubated with ligation-ligase solution at 37°C for 30 min, followed by incubation with amplification polymerase solution at 37°C for 100 min. Fluorescent signals were visualized under a confocal microscope (Zeiss). Each distinct fluorescent dot represents the close proximity of two interacting proteins within the cells.

### Molecular docking

The 3D crystal structures of HMGB1 and BECN1 were acquired from the UniProt database (https://www.uniprot.org/). HDOCK software was used for molecular docking analysis. The parameters were set as follows: grid spacing, 1.200; angle step, 15.00. The PDB files of HMGB1 and BECN1 were input, and the binding energy was quantified to evaluate the affinity between the HMGB1 and BECN1 proteins. The optimal conformations of HMGB1 and BECN1 were subsequently visualized via PyMOL software (v2.5.5).

### Dual-luciferase reporter gene assay

A dual-luciferase reporter gene assay was performed in accordance with the manufacturer’s instructions. The BECN1 dual-luciferase reporter gene vector (GM-MC-143625) was purchased from Genomeditech (Shanghai, China), and the luciferase assay kit (MF648-01) was purchased from Mei5 Biotechnology Co., Ltd. (Beijing, China).

### Chromatin immunoprecipitation assay

The ChIP assay was performed in accordance with the manufacturer’s instructions. The UCSC and JASPAR databases predicted ZNF460 binding sites (+1598 to +1631) in the promoter region of BECN1. A ChIP assay kit (CP0581, LABLEAD, Beijing, China) was used for the analysis. Using the following primers, we amplified this region from the immunoprecipitated DNA samples: forward 5’ -CAAGGCACCAGCGGATTCAC-3’ and reverse 5’ CTCAGTGTGGATGATGGAGTGTTG-3’.

### Patient data

Eighty pairs of CRC tissues and their corresponding paracancerous tissues were obtained from Yantaishan Hospital, an affiliated hospital of Binzhou Medical University. These patients did not receive systemic chemotherapy, targeted therapy, or immunotherapy before surgery. All patients signed an informed consent form, and all tissues were pathologically confirmed.

### Immunohistochemistry

Following previously described methods (16), the tissue samples were subjected to IHC staining and blind scoring. Two pathologists independently scored all the samples, with a maximum IHC score of 12.

### Statistical analyses

All the statistical analyses were performed via the GraphPad 9.0 (RRID: SCR_002798) statistical software package. The data are expressed as the means ± SDs of three independent experiments repeated at least three times. Significant differences between groups were determined via Student’s *t* test. A *P* value of < 0.05 was considered statistically significant in all the cases.

## Results

### HMGB1 was induced and released from the nucleus into the cytoplasm after irradiation in CRC cells

**Figure 1:**
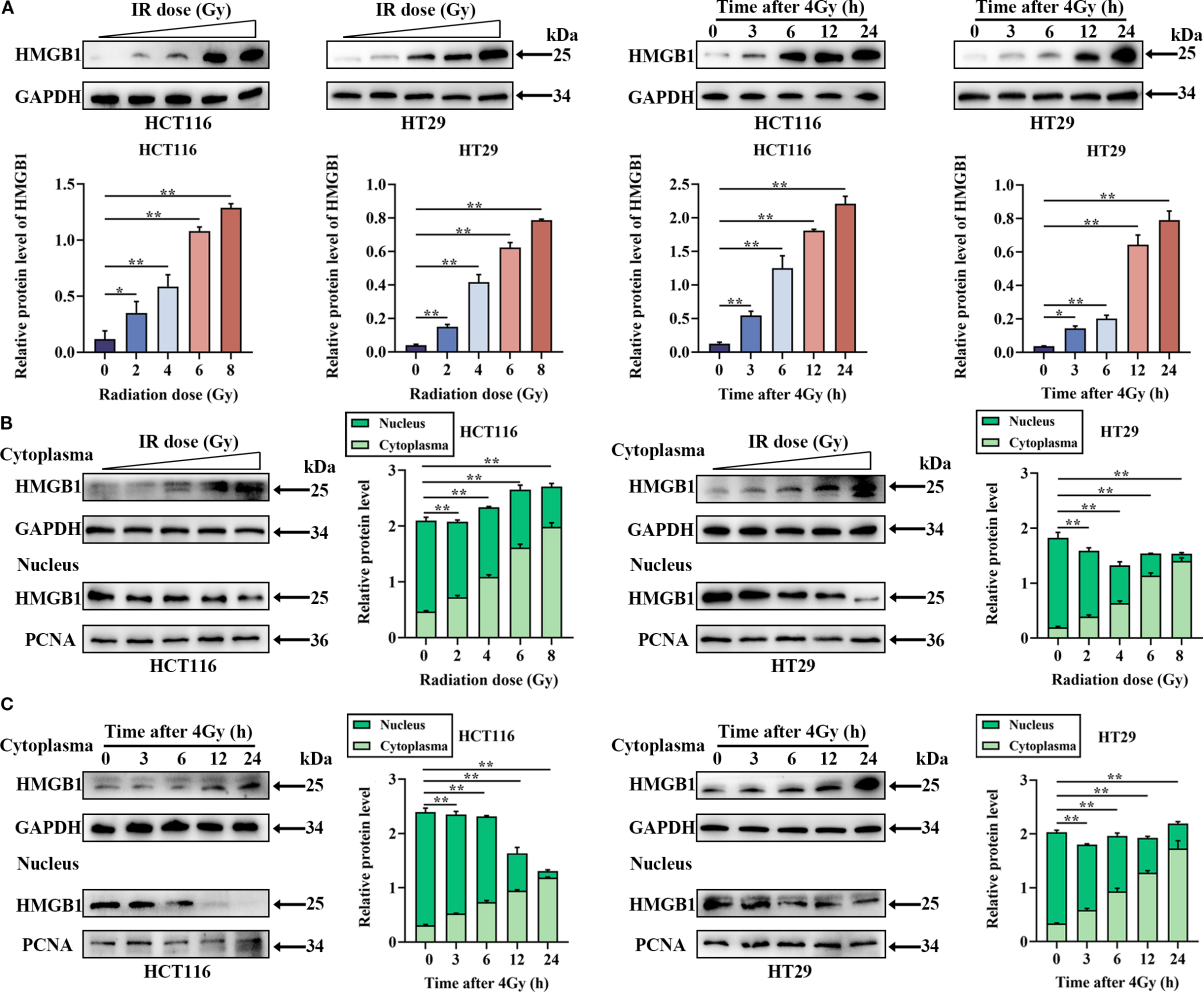
In CRC cells, HMGB1 is released into the cytoplasm from the nucleus after irradiation. **(A)** Radiation induced the expression of HMGB1 in CRC cells. Left panel, HMGB1 expression was assessed via western blotting after CRC cells were treated with different doses of irradiation (0, 2, 4, 6, or 8 Gy). Right panel, HMGB1 expression was assessed by western blotting at different time points (0, 3, 6, 12, and 24 h) following the exposure of CRC cells to 4 Gy X-ray irradiation. **(B)** Cell compartmentalization after exposure to different irradiation doses (0, 2, 4, 6, and 8 Gy). HMGB1 is localized in both the nucleus and cytoplasm of CRC cells. GAPDH and PCNA served as loading controls for the cytoplasmic and nuclear fractions, respectively. In the right panel, the relative densitometric units of HMGB1 were calculated. **(C)** Cell compartmentalization at different time points (0, 3, 6, 12, and 24 h) after 4 Gy irradiation revealed that HMGB1 was localized in both the nucleus and cytoplasm of CRC cells. GAPDH and PCNA were used as loading controls for the cytoplasmic and nuclear fractions, respectively. In the right panel, HMGB1 relative densitometry units were calculated. **P < 0.01, *P < 0.05.

To assess the expression of HMGB1 in CRC cells, we analyzed the mRNA and protein levels in CRC cells (HCT116, HT29, DLD-1, SW480, and SW620) and normal colonic epithelial cells (NCM460) via RT–qPCR and western blotting. As shown in **Supplementary Figure S1**, HMGB1 was more frequently upregulated in CRC cell lines than in NCM460 cells. Therefore, we selected the HCT116 and HT29 cells with the highest expression levels for subsequent experiments. Furthermore, to investigate the effect of irradiation on HMGB1 expression in CRC, we evaluated HMGB1 expression in CRC cells after ionizing radiation (IR). The results revealed that HMGB1 expression levels increased gradually after different doses of IR (0, 2, 4, 6, and 8 Gy) and gradually increased at different time intervals (0, 3, 6, 12, and 24 h) after 4 Gy irradiation (**Figure 1A**). In parallel, we analyzed the expression of HMGB1 in both the nucleus and cytoplasm of irradiated CRC cells at different time points after irradiation. Our results revealed that the expression of HMGB1 in the cytoplasm increased gradually, whereas the expression level of HMGB1 in the nucleus decreased gradually after treatment with different doses (**Figure 1B**). Similarly, translocation of HMGB1 from the nucleus to the cytoplasm was observed at different time intervals after 4 Gy irradiation (**Figure 1C**). These findings suggest that HMGB1 is induced and translocated from the nucleus to the cytoplasm after irradiation in CRC cells.

### HMGB1 knockdown enhanced the radiosensitivity of CRC *in vitro*

**Figure 2:**
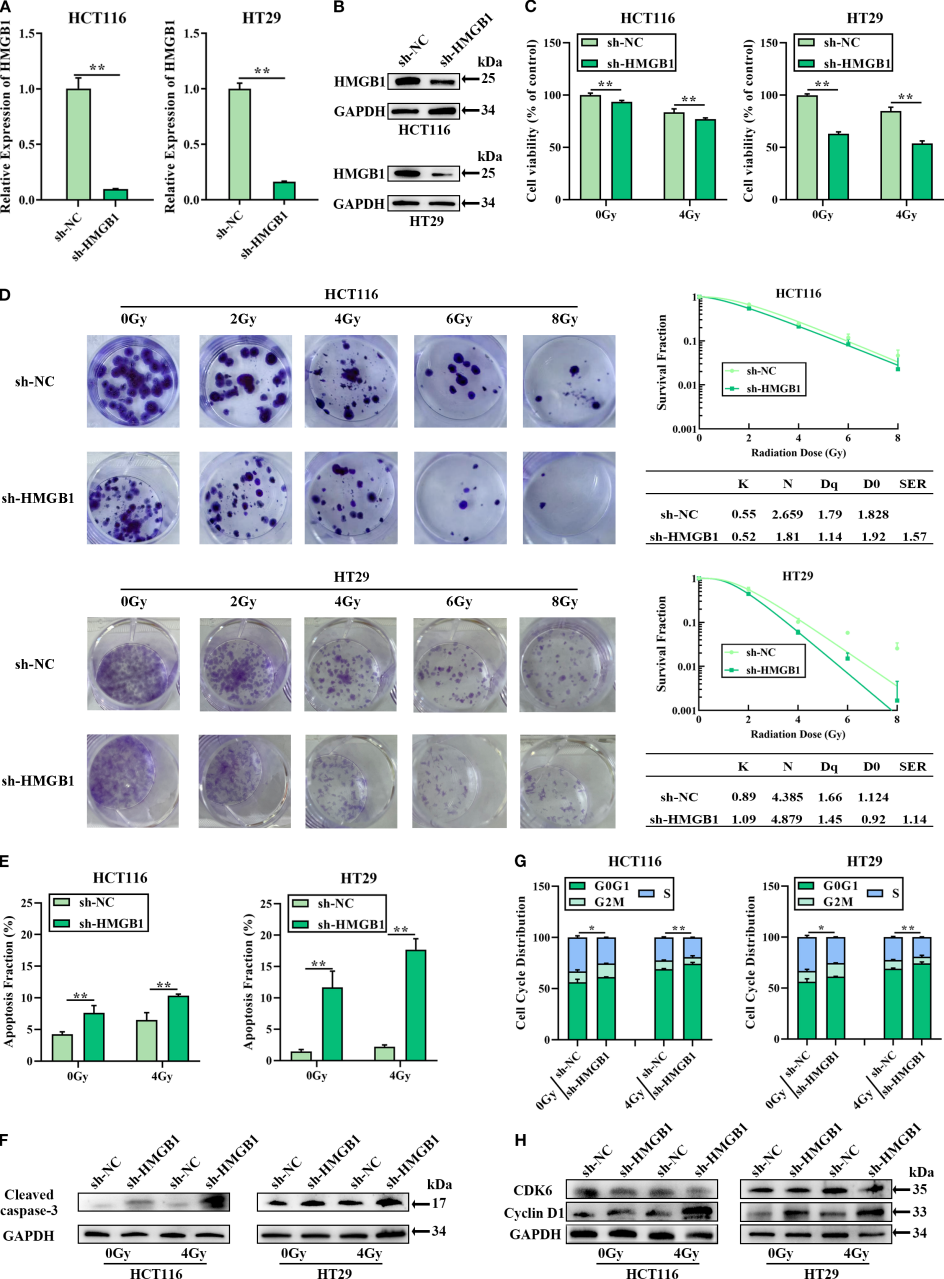
HMGB1 knockdown sensitized CRC cells to irradiation *in vitro*. **(A)** HMGB1 mRNA levels in both HCT116 and HT29 cells were analyzed via RT–qPCR after transfection with a siRNA negative control (sh-NC) or an HMGB1 siRNA (sh-HMGB1). **(B)** HMGB1 protein levels in CRC cells were analyzed by western blotting after transfection with sh-NC or sh-HMGB1, with GAPDH serving as the loading control. **(C)** The viability of HMGB1-depleted CRC cells after 4 Gy X-ray irradiation was assessed via CCK8 assays. **(D)** Cloning survival assays and survival curves of CRC cells after exposure to different irradiation doses (0, 2, 4, 6, and 8 Gy) were generated via GraphPad Prism 9.0 to measure radiosensitivity. **(E)** Apoptosis was measured via Annexin V/7-AAD double staining in HMGB1-depleted CRC cells after exposure to 4 Gy X-ray irradiation. **(F)** Protein expression of cleaved caspase 3 in HMGB1-depleted CRC cells after exposure to 4 Gy X-ray irradiation. GAPDH served as the loading control. **(G)** Effect of HMGB1 knockdown on the cell cycle distribution of HMGB1-depleted CRC cells after exposure to 4 Gy X-ray irradiation. **(H)** Protein expression of CDK6 and cyclin D1 in HMGB1-depleted CRC cells after exposure to 4 Gy X-ray irradiation. GAPDH served as the loading control. The values are expressed as the means ± SDs. **P < 0.01, *P < 0.05.

To investigate whether HMGB1 affects the biological function of CRC cells, HMGB1 depletion was performed via lentivirus in the CRC cell lines HCT116 and HT29. RT–qPCR and western blotting further confirmed that both the mRNA and protein levels of HMGB1 were significantly decreased (**Figures 2A, B**). After HMGB1 knockdown, we detected a significant reduction in CRC cell proliferation following IR via a CCK8 cell proliferation assay (**Figure 2C**). Consistent with the aforementioned results, the results of the colony formation assays revealed that HMGB1 knockdown markedly decreased the number and size of colonies after irradiation at different doses. In addition, the sensitization-enhancement ratios (SERs) of HCT116 and HT29 cells were 1.57 and 1.14, respectively (**Figure 2D**).

To investigate whether HMGB1-associated radioresistance is related to apoptosis and cell cycle progression, the apoptosis and cell cycle distribution of HMGB1-depleted CRC cells treated with 4 Gy X-ray irradiation were analyzed via flow cytometry. Compared with sh-NC, HMGB1 knockdown increased the number of apoptotic cells (**Figures 2E**; **Supplementary Figure S2A**). Furthermore, enhanced cleaved-caspase 3 expression was observed in HMGB1-depleted CRC cells after 4 Gy X-ray irradiation (**Figures 2F**; **Supplementary Figure S2B**). The cell cycle progression results revealed that the percentage of HMGB1-depleted CRC cells in the G0–G1 phase was significantly greater than that of sh-NC-treated cells after irradiation (**Figures 2G**; **Supplementary Figure S2C**). Moreover, the protein levels of cyclin D1 and CDK6, two cell cycle-associated genes, were examined in HMGB1-depleted CRC cells via western blotting. HMGB1 depletion induced the expression of cyclin D1 in CRC cells, whereas HMGB1 had no significant effect on CDK6 expression (**Figures 2H**; **Supplementary Figure S2D**). These results clearly demonstrate that HMGB1 protects CRC cells against irradiation.

### HMGB1 knockdown enhances the radiosensitivity of CRC *in vivo*

**Figure 3:**
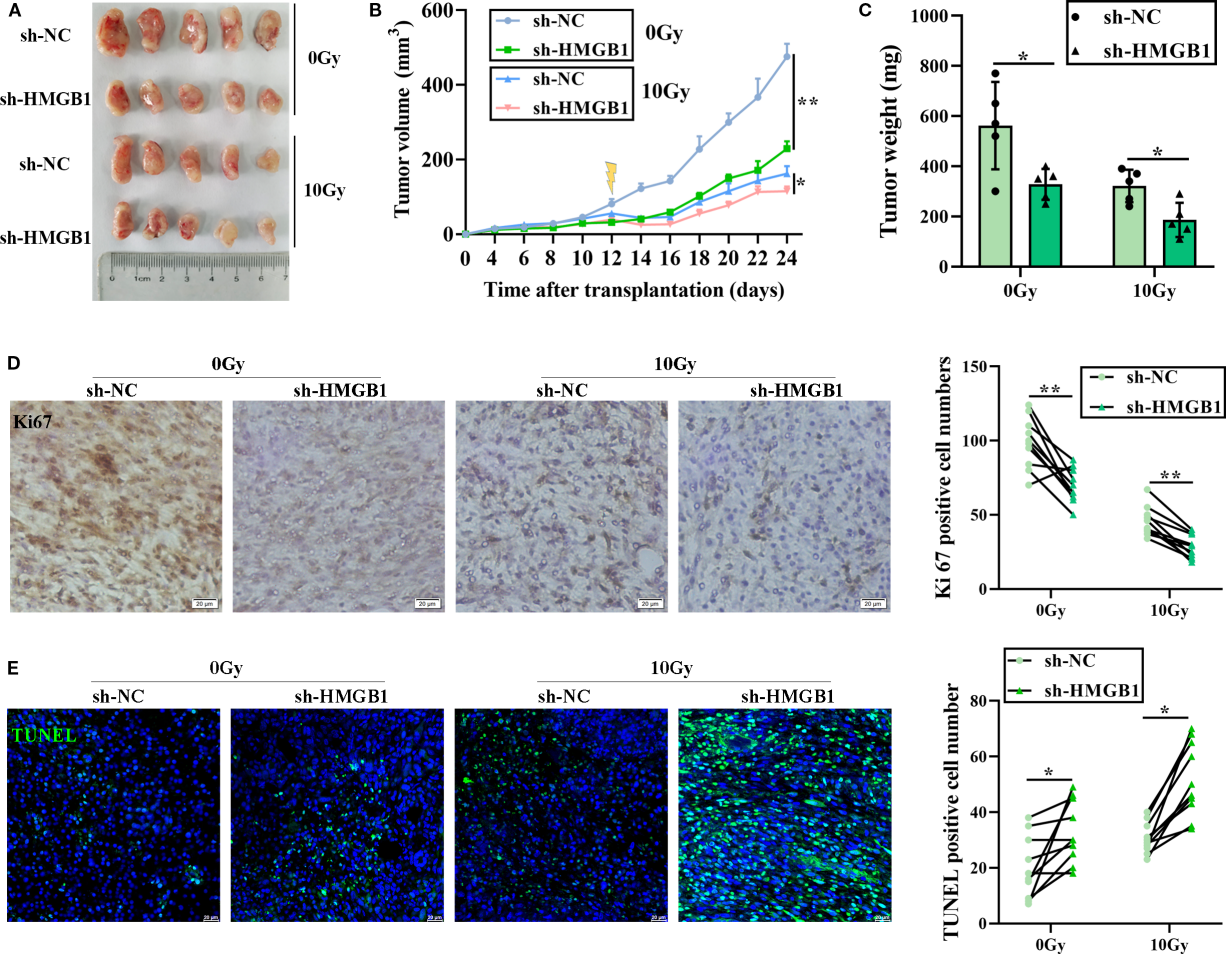
HMGB1 knockdown sensitized CRC cells to irradiation *in vivo*. **(A)** Each group comprised five female nude mice. Representative images of tumors formed by sh-NC or sh-HMGB1-HT29 cells treated with 10 Gy X-ray irradiation. **(B)** Growth curves of tumors formed by the indicated CRC cells treated with 10 Gy X-ray irradiation. **(C)** Weights of tumors formed by the indicated CRC cells treated with 10 Gy X-ray irradiation. The data are presented as the means ± SEMs (n = 5 mice per group). **(D)** Ki67 IHC staining of tumor tissues from the xenograft model with the indicated treatments (scale bar: 20 μm). The number of Ki67^+^ cells per visual field was quantified in 10 visual fields per group. **(E)** TUNEL staining of tumor tissues from the xenograft model with the indicated treatments (scale bar: 20 μm). The number of TUNEL^+^ cells per visual field was quantified in 10 visual fields per group. The data are presented as the means ± SEMs (n = 5 mice per group). **P < 0.01, *P < 0.05.

Given that HMGB1 has a key effect on CRC radiosensitivity *in vitro*, we further explored whether HMGB1 inhibits CRC cell proliferation *in vivo*. To this end, we constructed a subcutaneous xenograft tumor model of CRC in athymic nude mice, which included a sh-HMGB1 group and a control group (sh-NC), and the tumor-bearing mice were treated with 10 Gy X-ray irradiation. Compared with those in the sh-NC group, the tumors in the HMGB1 depletion and IR groups grew even slower in terms of tumor volume and weight (**Figures 3A–C**). Then, IHC was performed to examine the expression of the proliferation marker Ki67 in xenograft tumor tissues. As shown in **Figure 3D**, HMGB1 knockdown and irradiation significantly reduced the number of Ki67-positive cells in the xenografts. In contrast, the number of apoptotic cells in the xenograft tissues was also measured via the TUNEL assay. The number of apoptotic cells in the xenograft tissues was significantly increased in the sh-HMGB1 group under 10 Gy X-ray irradiation (**Figure 3E**). Together with the findings of our *in vitro* experiments, these results proved that HMGB1 knockdown sensitized CRC cell lines to IR and that an HMGB1 inhibitor could be developed as a therapeutic drug to increase the effect of radiotherapy in patients with CRC.

### HMGB1 combines with BECN1 in the cytoplasm of CRC cells after irradiation

Previous studies reported that IR can induce autophagy, leading to radioresistance, and that HMGB1 can promote autophagy by combining with BECN1, a key regulatory factor in autophagy (13, 18). Therefore, we hypothesized that HMGB1 knockdown may increase radiosensitivity in CRC by regulating autophagy. The following hypotheses can be tested: first, by investigating whether HMGB1 induces autophagy after IR in CRC cells; second, by investigating whether BECN1 translocates to the cytoplasm after IR; and finally, by determining whether HMGB1 promotes autophagy by binding to BECN1 in the cytoplasm of CRC cells.

**Figure 4:**
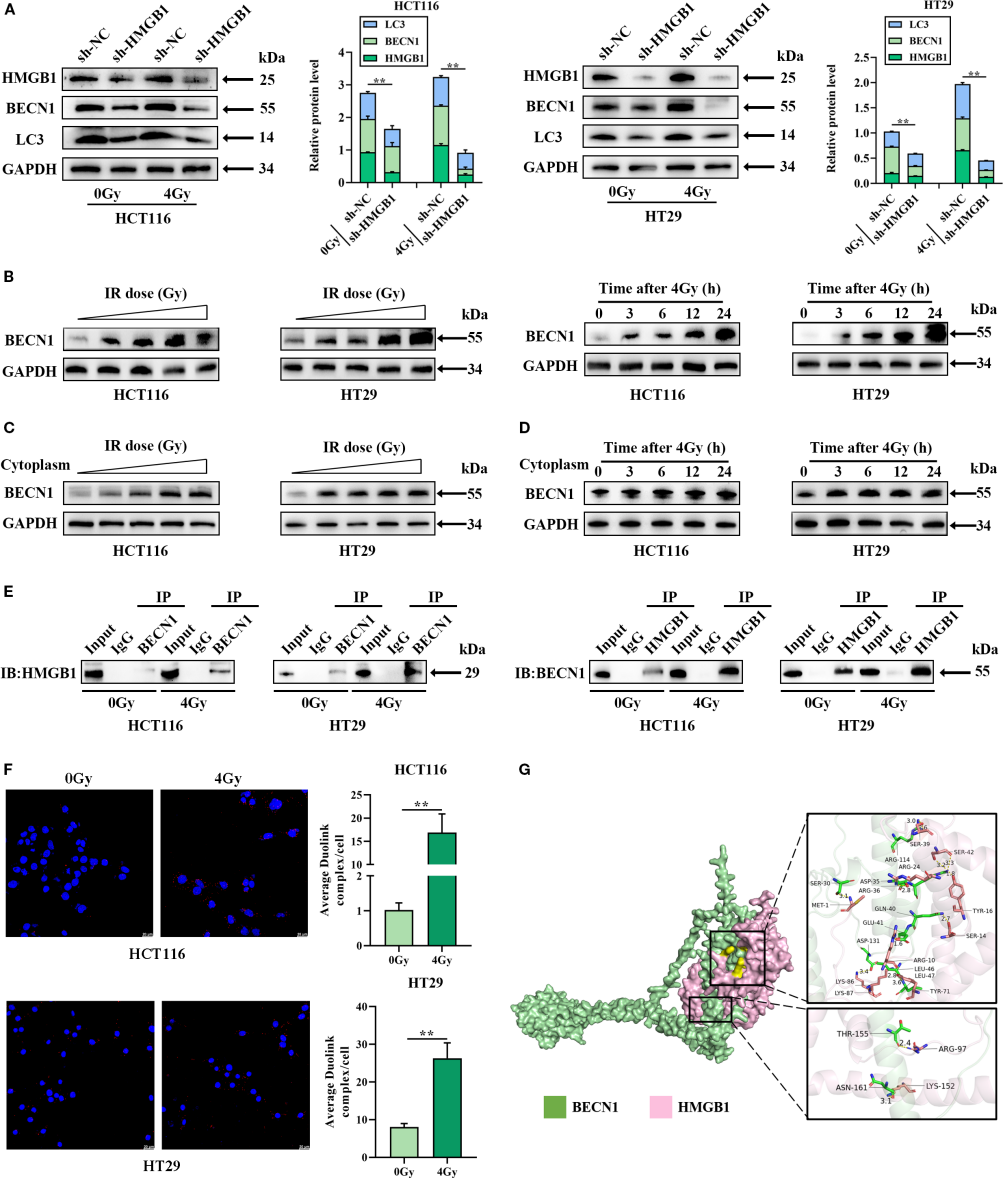
HMGB1 knockdown inhibits autophagy by binding to BECN1 in the cytoplasm of CRC cells. **(A)** Protein expression of HMGB1, BECN1, and LC3 in HMGB1-depleted CRC cells after exposure to 4 Gy X-ray irradiation. GAPDH served as the loading control. **(B)** Irradiation induced the expression of BECN1. Left panel: BECN1 expression was assessed by western blotting following treatment with different doses of irradiation (0, 2, 4, 6, and 8 Gy). Right panel, BECN1 expression was assessed by western blotting at different time points (0, 3, 6, 12, and 24 h) following exposure to 4 Gy X-ray irradiation. **(C)** Protein expression of BECN1 in the cytoplasm of CRC cells after exposure to different irradiation doses (0, 2, 4, 6, and 8 Gy). GAPDH served as the loading control. **(D)** Protein expression of BECN1 in the cytoplasm of CRC cells at different time points (0, 3, 6, 12, and 24 h) following exposure to 4 Gy X-ray irradiation. GAPDH served as the loading control. **(E)** Coimmunoprecipitation analysis was performed to evaluate the formation of the HMGB1 and BECN1 immune complex. **(F)** PLA was performed to evaluate the formation and location of the HMGB1 and BECN1 immune complex in CRC cells. **(G)** Molecular docking model of HMGB1 and BECN1, and the zoomed-in image illustrates the interaction of HMGB1 with amino acid residues near the binding site. **P < 0.01, *P < 0.05.

We tested whether HMGB1 promotes autophagy by measuring the levels of BECN1 and LC3 after irradiation via western blotting. The results revealed that 4 Gy X-ray irradiation induced BENC1 and LC3 protein expression in CRC cells, whereas HMGB1 knockdown decreased BENC1 and LC3 protein levels (**Figure 4A**). We also tested whether BECN1 was translocated to the cytoplasm after IR in CRC cells. The results of western blotting revealed that BECN1 expression increased gradually at different concentrations (0, 2, 4, 6, and 8 Gy). The expression of BECN1 also gradually increased at different time intervals (0, 3, 6, 12, and 24 h) after 4 Gy irradiation (**Figure 4B**; **Supplementary Figure S3A**). The results revealed that the expression of BECN1 in the cytoplasm increased gradually with increasing radiation dose and time (**Figures 4C, D**; **Supplementary Figures S3B, C**).

Finally, we investigated whether HMGB1 combines with BECN1 in the cytoplasm of CRC cells via co-IP and a Duolink *in situ* proximity ligation assay (PLA). As shown in **Figure 4E**, HMGB1 binding to BECN1 was stronger after irradiation, whereas the interaction between BECN1 and HMGB1 was weaker than that between HMGB1 and BECN1 according to the Co-IP assay. Furthermore, the PLA revealed a stronger combination of HMGB1 with BECN1 in the cytoplasm of CRC cells after 4 Gy X-ray irradiation (**Figure 4F**). Using molecular docking analysis to evaluate the binding affinity of BECN1 for HMGB1, we characterized it as having a low binding energy of -314.03 kcal/mol (**Figure 4G**). These results showed that HMGB1 promoted autophagy by binding to BECN1 in CRC after IR treatment.

### HMGB1 promoted the release of BECN1 by ZNF460 in CRC cells after irradiation

**Figure 5:**
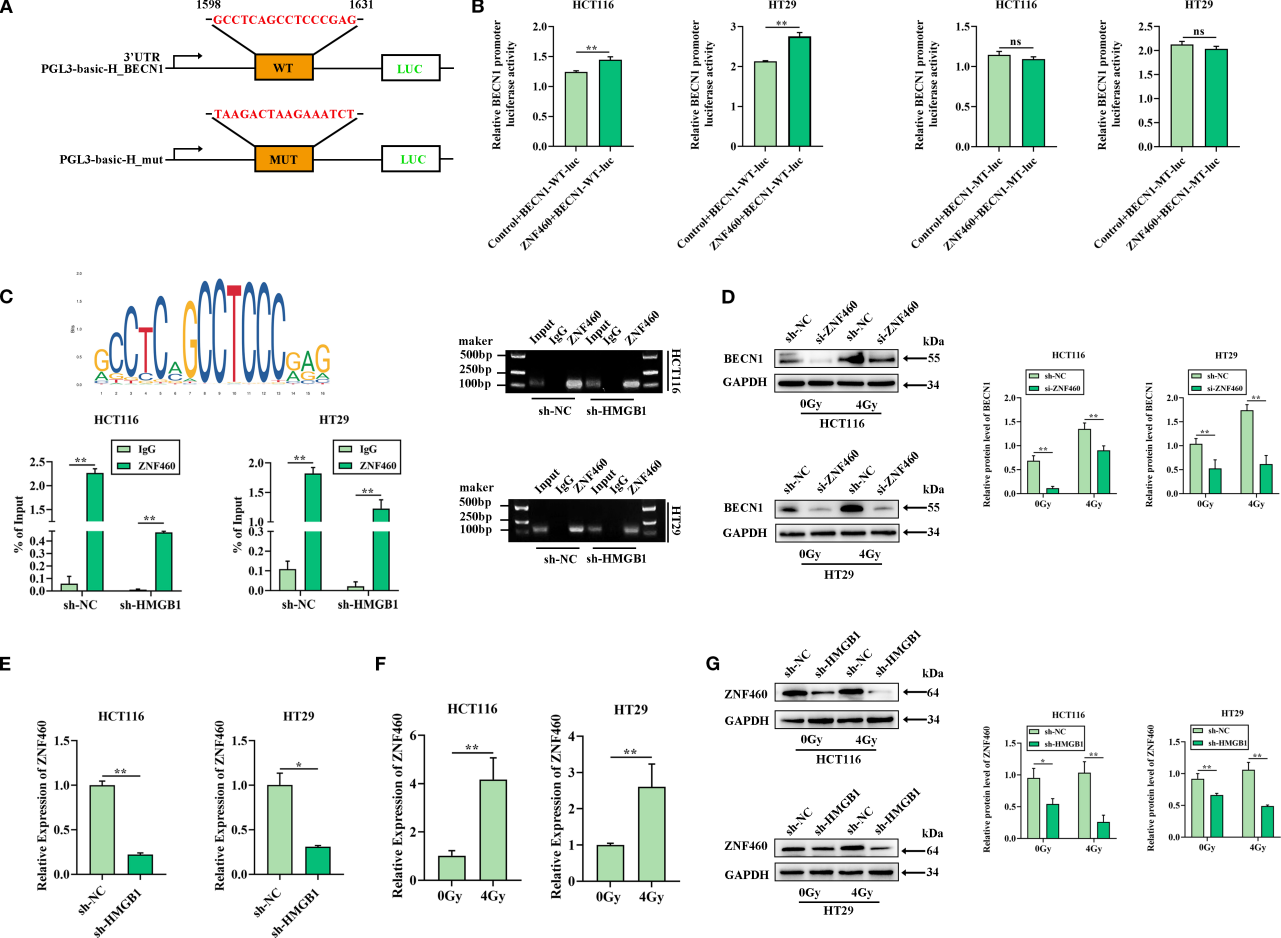
HMGB1 enhances the release of BECN1 by ZNF460 in CRC cells after irradiation. **(A)** The JASPAR website was used to scan the BECN1-bound promoter and predict one potential binding site (GCCTCAGCCTCCGAG) for BECN1. The relative score (RS) = 0.90 for the motifs. **(B)** A dual-luciferase reporter showed that ZNF460 increased the luciferase activity of the reporter carrying the wild-type 3′ UTR fragment of BECN1 (left panel), whereas these changes were abolished when the binding sites were mutated in CRC cells (right panel). **(C)** Enrichment of BECN1 promoter fragments via an antibody against ZNF460 was assessed through ChIP–qPCR analysis. Agarose electrophoresis analysis of PCR products from the BECN1 promoter after ChIP–PCR was also conducted. **(D)** Protein expression of BECN1 in ZNF460-depleted CRC cells after exposure to 4 Gy X-ray irradiation. GAPDH served as the loading control. **(E)** ZNF460 mRNA levels in both sh-HCT116 and sh-HT29 cells were analyzed via RT–qPCR. **(F)** ZNF460 mRNA levels in both HCT116 and HT29 cells were analyzed after exposure to 4 Gy X-ray irradiation. **(G)** Protein expression of ZNF460 in HMGB1-depleted CRC cells after exposure to 4 Gy X-ray irradiation. GAPDH served as the loading control. **P < 0.01, *P < 0.05.

To investigate the release of BECN1 in CRC cells after irradiation, the UCSC and JASPAR databases were used to predict the transcription factor ZNF460, which can bind to the BECN1 promoter. To verify the interaction between ZNF460 and the 3′UTR of BECN1, luciferase reporter assays were performed using the wild-type BECN1 3′UTR sequence, which contains either the seed sequence for ZNF460 recognition (BECN1-3′UTRwt) or a mutated 3′UTR (BECN1-3′UTRmut) (**Figure 5A**). The relative luciferase activity of the BECN1-3′UTRwt reporter was significantly reduced by ZNF460 relative to that of the NC mimics; however, mutation of the ZNF460 seed sequence abolished this inhibitory effect (**Figure 5B**). Moreover, ChIP assays confirmed that ZNF460 bound directly to the BECN1 promoter region (**Figure 5C**). To further investigate the regulation of BECN1 release by ZNF460, a siRNA targeting ZNF460 was used, which reduced the expression of ZNF460 in CRC cells (**Supplementary Figure S3D**), and the western blotting results revealed that BECN1 was significantly reduced after ZNF460 knockdown after irradiation in CRC cells (**Figure 5D**). These results showed that ZNF460 could directly bind to the BECN1 promoter, thereby promoting the release of BECN1.

We further explored whether HMGB1 and radiation regulate the expression of ZNF460. The RT–qPCR results revealed that, compared with sh-NC, HMGB1 knockdown decreased ZNF460 expression (**Figure 5E**). Furthermore, ZNF460 expression was increased in CRC cells after 4 Gy X-ray irradiation (**Figure 5F**). In addition, HMGB1 knockdown markedly reduced the protein level of ZNF460 (**Figure 5G**). Overall, these results indicated that HMGB1 promoted the release of BECN1 through ZNF460 after irradiation in CRC.

### The HMGB1/BECN1 complex confers radioresistance *in vitro* and *in vivo*

**Figure 6:**
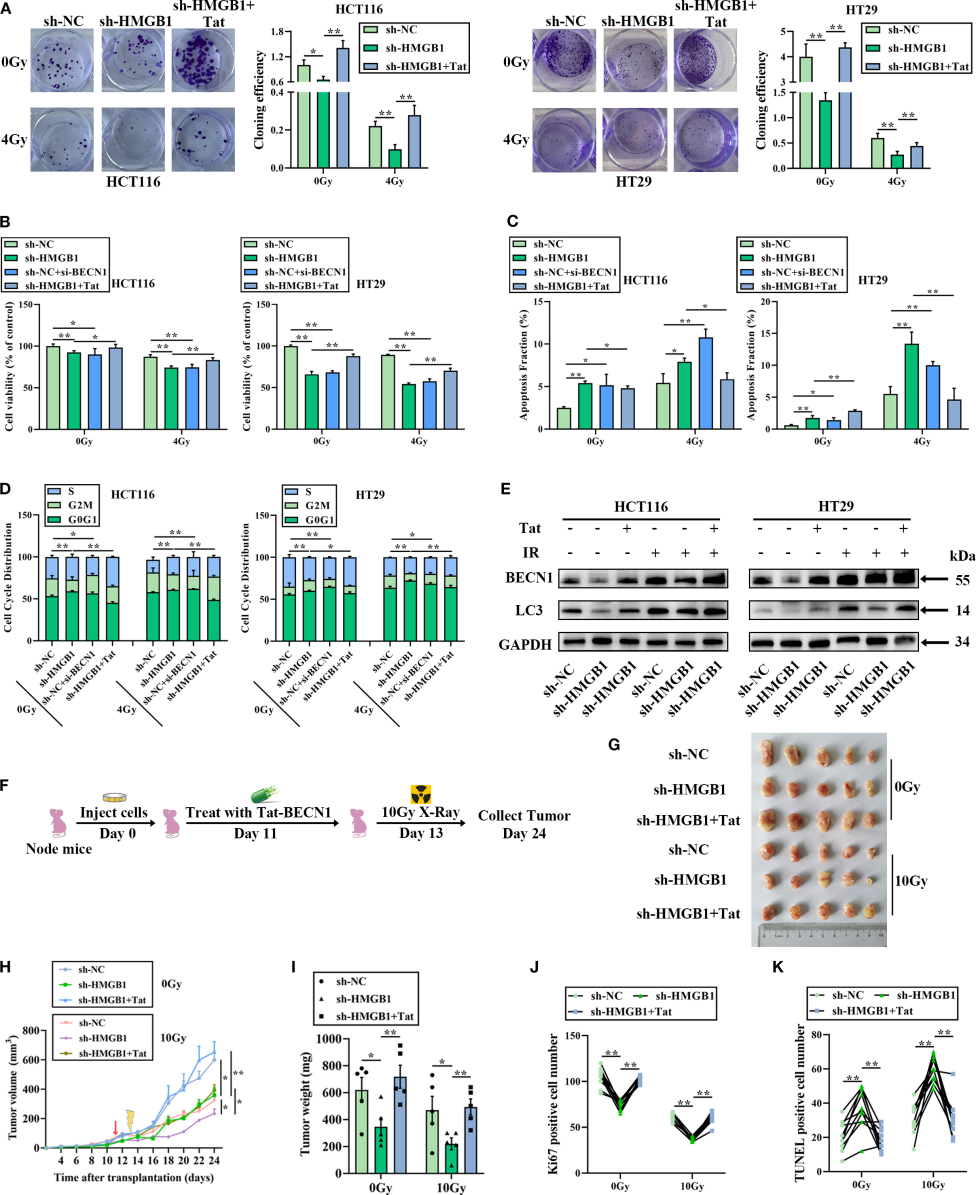
The HMGB1/BECN1 axis confers resistance to irradiation both *in vitro* and *in vivo*. **(A)** Colony formation was assessed in HMGB1-depleted CRC cells treated with BECN1 siRNA (si-BECN1) or Tat-BECN1 (Tat) before 4 Gy X-ray irradiation. **(B)** Viability was assessed in HMGB1-depleted CRC cells treated with si-BECN1 or Tat before 4 Gy X-ray irradiation. **(C)** Apoptosis was measured via Annexin V/7-AAD double staining in HMGB1-depleted CRC cells treated with si-BECN1 or Tat before 4 Gy X-ray irradiation. **(D)** Cell cycle progression was measured in HMGB1-depleted CRC cells treated with si-BECN1 or Tat before 4 Gy X-ray irradiation. **(E)** Protein expression of BECN1 and LC3 in HMGB1-depleted CRC cells treated with Tat before 4 Gy X-ray irradiation. GAPDH served as the loading control. **(F)** The treatment regimen used in each group is shown. HT29 cells were inoculated under the skin of nude mice. On day 11 after translation, 5 mg/kg Tat was intraperitoneally injected into the nude mice. On day 13, the xenograft mice were locally irradiated with 10 Gy X-rays. The tumor size was measured at 2-day intervals. **(G)** Each group comprised five female nude mice. Representative images of tumors formed by sh-NC or sh-HMGB1-HT29 cells treated with Tat and 10 Gy X-ray irradiation. **(H)** Growth curves of tumors formed by the indicated CRC cells treated with Tat and 10 Gy X-ray irradiation. **(I)** Weights of tumors formed by the indicated CRC cells treated with Tat and 10 Gy X-ray irradiation. The data are presented as the means ± SEMs (n = 5 mice per group). **(J)** Ki67-positive cell numbers in the tumor tissues of the nude mouse xenograft model treated with the indicated drugs. **(K)** TUNEL-positive cell numbers in the tumor tissues of the nude mouse xenograft model treated with the indicated drugs. The data are presented as the means ± SEMs (n = 5 mice per group). **P < 0.01, *P < 0.05.

We hypothesized that HMGB1 contributes to radioresistance by combining with BECN1 in CRC cells. To test this hypothesis, a siRNA targeting BECN1, which reduces the expression of BECN1 in CRC cells (**Supplementary Figures S4A, B**), or Tat-BECN1 (Tat), a BECN1 activator, was used to treat HMGB1-depleted CRC cells. The IC50 values of Tat-BECN1 in CRC cells were 5.010 and 5.042 μM in HCT116 and HT29 cells, respectively (**Supplementary Figure S4C**). Therefore, 5 μM was selected for the subsequent experiments. Colony formation assays revealed that HMGB1 or BECN1 deletion significantly decreased the number and size of CRC cells, whereas Tat-BECN1 treatment increased colony formation in HMGB1-depleted CRC cells (**Figure 6A**). The results of the cell viability assays revealed that HMGB1 or BECN1 knockdown clearly reduced cell proliferation, whereas Tat-BECN1 treatment abolished HMGB1 knockdown-induced radiosensitivity (**Figure 6B**). In addition, HMGB1 or BECN1 knockdown significantly increased apoptosis, whereas Tat-BECN1 treatment noticeably decreased the apoptotic population and the level of cleaved caspase 3 in HMGB1-depleted CRC cells (**Figures 6C**; **Supplementary Figures S4D, E**). Furthermore, the cell cycle distribution results revealed that HMGB1 or BECN1 knockdown increased G0G1-phase arrest, whereas Tat-BECN1 treatment significantly decreased the percentage of G0G1-phase HMGB1-depleted CRC cells (**Figures 6D**; **Supplementary Figure S5A**). Moreover, Tat-BECN1 treatment decreased the protein level of cyclin D1 in HMGB1-depleted CRC cells, whereas Tat had no significant effect on CDK6 expression (**Supplementary Figure S5B**). In contrast, Tat-BECN1 treatment abolished the inhibitory effect of HMGB1 knockdown on autophagy (**Figures 6E**; **Supplementary Figure S5C**). These findings reveal that HMGB1-induced autophagy by binding with BECN1 contributes to the radioresistance of CRC *in vitro*.

To further elucidate the effect of Tat-BECN1 in an animal model, HMGB1 knockout and negative control HT29 cells were injected into the subcutaneous tissues of nude mice to establish a xenograft model. Starting 4 days after tumor formation, the mice were randomly divided into two groups. One group received an intraperitoneal injection of Tat-BECN1, while the other group was treated with 10 Gy X-ray irradiation at the tumor site (**Figure 6F**). Histological analysis of tumor sections revealed that HMGB1 knockdown with IR significantly reduced the tumor volume and weight, whereas Tat-BECN1 treatment obviously increased tumor growth and weight (**Figures 6G–I**), in accordance with the *in vitro* data described above. IHC analysis revealed that HMGB1 deletion significantly reduced the number of Ki67-positive subcutaneous tumors and increased the number of apoptotic cells in the xenografts after 10 Gy X-ray irradiation. In contrast, Tat-BECN1 treatment significantly promoted cell proliferation and reduced apoptosis (**Figures 6J, K**; **Supplementary Figures S5D, E**). Taken together, these data indicate that HMGB1-induced radioresistance is due to increased levels of autophagy when HMGB1 is combined with BECN1.

### HMGB1 and BECN1 overexpression is associated with poor prognosis in patients with CRC

**Figure 7:**
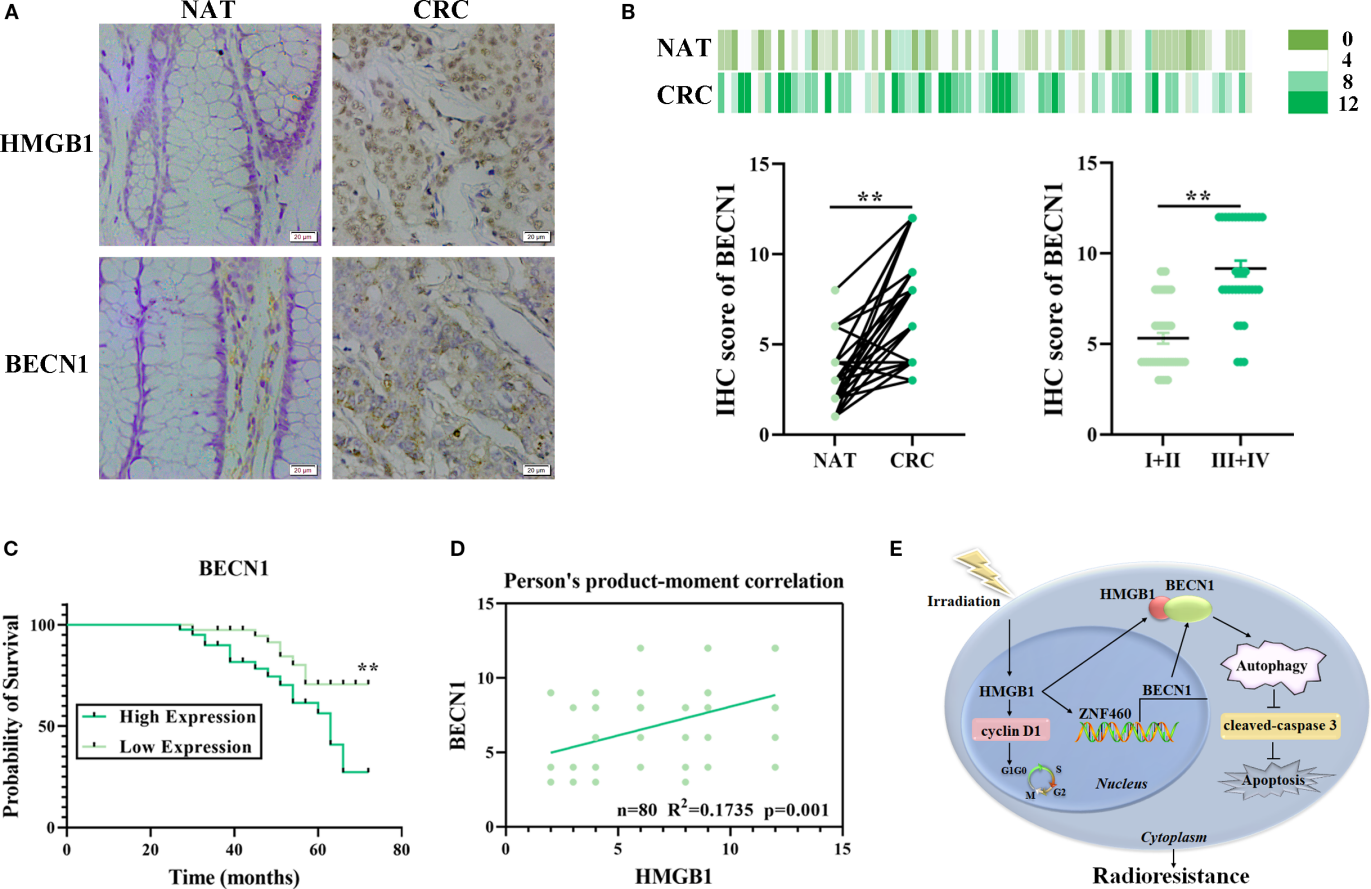
Correlation analysis of HMGB1 and BECN1 expression in CRC tissue samples. **(A)** Representative image of the IHC analysis of HMGB1 and BECN1 protein expression in CRC (n = 80) tissue sections at different stages (scale bar: 20 µm). **(B)** BECN1 protein expression based on the staining index in nonmalignant adjacent tissues (NATs) and CRC samples at different clinical stages. The values are expressed as the means ± SEMs. **(C)** Kaplan–Meier curves of overall survival according to BECN1 expression in patients with CRC. **(D)** Correlation analysis of the staining indices of the protein expression levels of HMGB1 and BECN1 in human CRC samples (n = 80). **(E)** Schematic illustration of how HMGB1 enhances the radioresistance of CRC. Radiation causes HMGB1 to be released into the cytoplasm. HMGB1 knockout inhibits autophagy by reducing the release and binding of BECN1 in the cytoplasm via ZNF460, thereby promoting cell cycle arrest and apoptosis and ultimately increasing radiosensitivity. **P < 0.01, *P < 0.05.

Our previous study revealed that HMGB1 was abnormally expressed and associated with poor prognosis in patients with CRC (9). To further explore the relationships among HMGB1, BECN1, and the clinicopathological features of patients with CRC, we used IHC to analyze tissue microarrays containing 80 CRC and adjacent normal (NAT) tissues. The clinicopathological features of the 80 patients are presented in **Supplementary Table S3**. The results demonstrated that the protein expression levels of both HMGB1 and BECN1 were significantly greater in CRC tissues than in adjacent normal tissues (**Figure 7A**). In addition, we observed that HMGB1 and BECN1 expression increased with tumor stage. The BECN1 levels were higher in advanced clinical stages (III and IV) than in early stages (I and II) (**Figures 7B**; **Supplementary Table S3**). We next investigated the correlation between BECN1 levels and survival outcomes in patients with CRC. According to the median expression levels of BECN1, we divided the 80 patients with CRC into a low-BECN1-expression group (n = 39) and a high-BECN1-expression group (n = 41). Consistently, patients with higher BECN1 expression had worse overall survival (**Figure 7C**). Pearson analysis revealed a positive correlation between the levels of HMGB1 and BECN1 in patients with CRC (**Figure 7D**). These results indicate that HMGB1 and BECN1 are frequently highly regulated in patients, highlighting the potential role of HMGB1 in CRC progression.

## Discussion

Although refinements in surgery, including the acceptance of total mesorectal excision, modern chemotherapy, and advances in the timing and dosimetry of radiotherapy, have significantly affected local tumor control, distant relapse remains the leading cause of mortality in patients with CRC (19). Thus, identifying radiosensitive therapy markers has important clinical implications, as it will improve the outcomes of radiotherapy-based treatment regimens for these patients. Emerging evidence suggests that HMGB1 plays a role in CRC. Meng et al. reported that MSI2 improved the prognosis of CRC patients by reprogramming the tumor immune microenvironment through HMGB1-mediated posttranslational modifications (20). Moreover, IHC analysis revealed that HMGB1 cytoplasmic translocation was associated with poor prognosis in CRC patients (21). However, few studies have investigated the role and mechanism of HMGB1 in the radiosensitivity of CRC. Herein, we observed that HMGB1 promotes the proliferation of colorectal cancer cells and enhances their resistance to radiation, suggesting that HMGB1 directly regulates the radioresistance of tumor cells. Additionally, HMGB1 may act as a molecular decoy by recruiting ZNF460 to increase BECN1 release, which in turn promotes ZNF460 transcription and activates the BECN1-mediated autophagy pathway. Therefore, HMGB1 plays a role in CRC radioresistance via the autophagy pathway (**Figure 7E**).

In the present study, we found that HMGB1 is expressed in both the cytoplasm and nucleus and that irradiation not only increased the expression of total HMGB1 protein but also increased the translocation of HMGB1 into the cytoplasm. Multiple studies have illustrated the different roles of HMGB1 in different locations of cells. Lou et al. demonstrated that the long noncoding RNA BS-DRL1 modulates the DNA damage response and genome stability by interacting with HMGB1 in neurons (22). Cui et al. reported that cytoplasmic circHERC1 facilitates the invasion and metastasis of NSCLC cells by regulating the miR-142-3p/HMGB1 axis and activating the MAPK/ERK and NF-κB pathways (23). Although He et al. reported that HMGB1 does not change significantly in the nucleus or cytoplasm after 10 Gy irradiation (24), Zhang et al. reported the cytoplasmic translocation of HMGB1 in irradiated neurons (25). These findings are consistent with our results, indicating that cytoplasmic HMGB1 can contribute to tumor radioresistance. We discovered that HMGB1 has a noticeable effect on apoptosis and autophagy in CRC cells after irradiation, as evidenced by changes in characteristic proteins, such as cleaved caspase 3, BECN1 and LC3, upon alterations in HMGB1 expression. Taken together, our data strongly support the notion that HMGB1 promotes cell proliferation and autophagy in CRC cells after irradiation, indicating that the knockout of HMGB1 in combination with radiotherapy can more effectively induce autophagy in cells, resulting in the killing of tumor cells. This outcome promotes the clinical implications of our experiment.

Additionally, our results revealed that the autophagy pathway in CRC cells is regulated by the release of BECN1 mediated by HMGB1. Our experiments revealed a direct interaction between BECN1 and HMGB1 in the cytoplasm, resulting in its activity. As a key nonhistone DNA-binding protein, HMGB1 is critical for the modulation of autophagy in multiple types of cancer cells, as outlined by many studies. Chen et al. reported that lucidone treatment enhances chemosensitivity by inhibiting the expression of autophagic proteins (Atg5, Beclin-1, LC3-II, and Vps34) via the inhibition of the HMGB1/RAGE/PI3K/Akt axis in pancreatic cancer cells (26). Recent studies have shown that HMGB1 promotes chemotherapy resistance in breast cancer and esophageal cancer through HMGB1-mediated autophagy (27, 28). Importantly, the inhibition of autophagy can increase the radiosensitivity of several types of cancer (29, 30). In this study, we found that the total and cytoplasmic protein levels of BECN1 increased with increasing radiation dose and that BECN1 expression increased with time after irradiation. In addition, Min et al. reported that the cytosolic HMGB1–BECN1 complex increased radioresistance in oral squamous cell carcinoma (14). Indeed, we observed that HMGB1 is translocated outside the nucleus, where it interacts with BECN1 in the cytoplasm of radioresistant CRC cells, thereby increasing autophagic flux. Importantly, the increase in radiosensitivity was abolished by inducing autophagy with Tat-BECN1 treatment both *in vitro* and *in vivo*. More importantly, there was a positive correlation between HMGB1 and BECN1 levels in patients with CRC. On the other hand, correlations between HMGB1 and BECN1 and their associations with TNM stage and prognosis were also demonstrated in CRC clinical samples. Therefore, the HMGB1/BECN1 axis, which is critical for colorectal cancer progression, can represent a therapeutic marker for patients with CRC, particularly those with radioresistance. Unfortunately, we have not focused on alternative autophagy pathways contributing to radioresistance, which will be explored in future studies.

Furthermore, we also found that the release of BECN1 in CRC cells is regulated by the transcription of ZNF460, which is mediated by HMGB1. Our experiments demonstrated a direct interaction between ZNF460 and the BECN1 promoter in the region from +1598–+1631, thereby impacting BECN1-mediated autophagy activity. Previous studies have shown that some ZNFs have multiple effects on promoting tumor proliferation, migration and invasion. Hao et al. demonstrated that hyperexpression of ZNF460 was associated with adverse prognosis and that it facilitated cell migration in colon cancer via the JAK2/STAT3 signaling pathway (31). An et al. reported that ZNF460 mediated epithelial–mesenchymal transition to promote gastric cancer progression (32). In addition, HMGB1 and irradiation were found to induce the expression of ZNF460, suggesting that HMGB1 promotes the release of BECN1 into the cytoplasm by ZNF460 in CRC cells after irradiation. Taken together, these results suggest that high HMGB1 expression is expected to promote CRC radioresistance by increasing autophagy via the promotion and binding of BECN1 via ZNF460.

### Study strengths and limitations

Tumor resistance to radiotherapy arises not only from genetic mutations within cancer cells but also from the continuous interaction and co-evolution between cancer cells and their surrounding microenvironment (33, 34). Within this “ecological-evolutionary” framework, the study investigates the role of cytoplasmic HMGB1 in enhancing autophagy and promoting radioresistance in CRC through direct interaction with BECN1 and transcriptional regulation of ZNF460, identifying the HMGB1/ZNF460/BECN1 axis as a potentially actionable molecular target. Nevertheless, the study has several limitations, including the relatively small clinical cohort (n=80), the use of subcutaneous xenograft models that may not fully recapitulate the complexity of the human tumor microenvironment, and the lack of exploration into alternative autophagy pathways that may contribute to radioresistance. Additionally, the absence of long-term efficacy assessments and potential variations in radiation dosing across experimental setups may affect the generalizability and reproducibility of the findings. Future research aims to incorporate patient-derived organoids, spatial transcriptomic analyses, and targeted microenvironmental interventions. These approaches are expected to further contextualize the molecular insights regarding the HMGB1/ZNF460/BECN1 axis within the broader “tumor ecosystem” framework, thereby offering a more comprehensive strategy to address radiotherapy resistance in CRC.

## Conclusions

In summary, we demonstrated that HMGB1 can enhance radioresistance by promoting cell proliferation and autophagy in CRC cells both *in vitro* and *in vivo*. This process is coregulated by BECN1 and ZNF460. HMGB1 not only promotes the release of BECN1 via ZNF460 but also directly binds to BECN1 in the cytoplasm, thereby activating autophagy and ultimately influencing the effectiveness of HMGB1 in response to radiotherapy. Importantly, our results from human CRC specimens indicated that HMGB1 was positively correlated with BECN1 expression and significantly associated with poor prognosis. Our study revealed that the HMGB1/ZNF460/BECN1 axis contributes to radioresistance and autophagy in CRC, suggesting that HMGB1 could be a potential therapeutic target for enhancing the response of CRC to radiation therapy.

## Data Availability

The original contributions presented in the study are included in the article/**Supplementary Material**. Further inquiries can be directed to the corresponding author.
