# Supplementary material for: Cytoplasmic HMGB1 promotes and interacts with BECN1 through ZNF460 to induce autophagy and accelerate radioresistance in colorectal cancer cells
Source: Front Immunol. 2025 Oct 14;16:1642915. doi: 10.3389/fimmu.2025.1642915 (PMC12558843; doi:10.3389/fimmu.2025.1642915)

Full length WB to Fig 1A

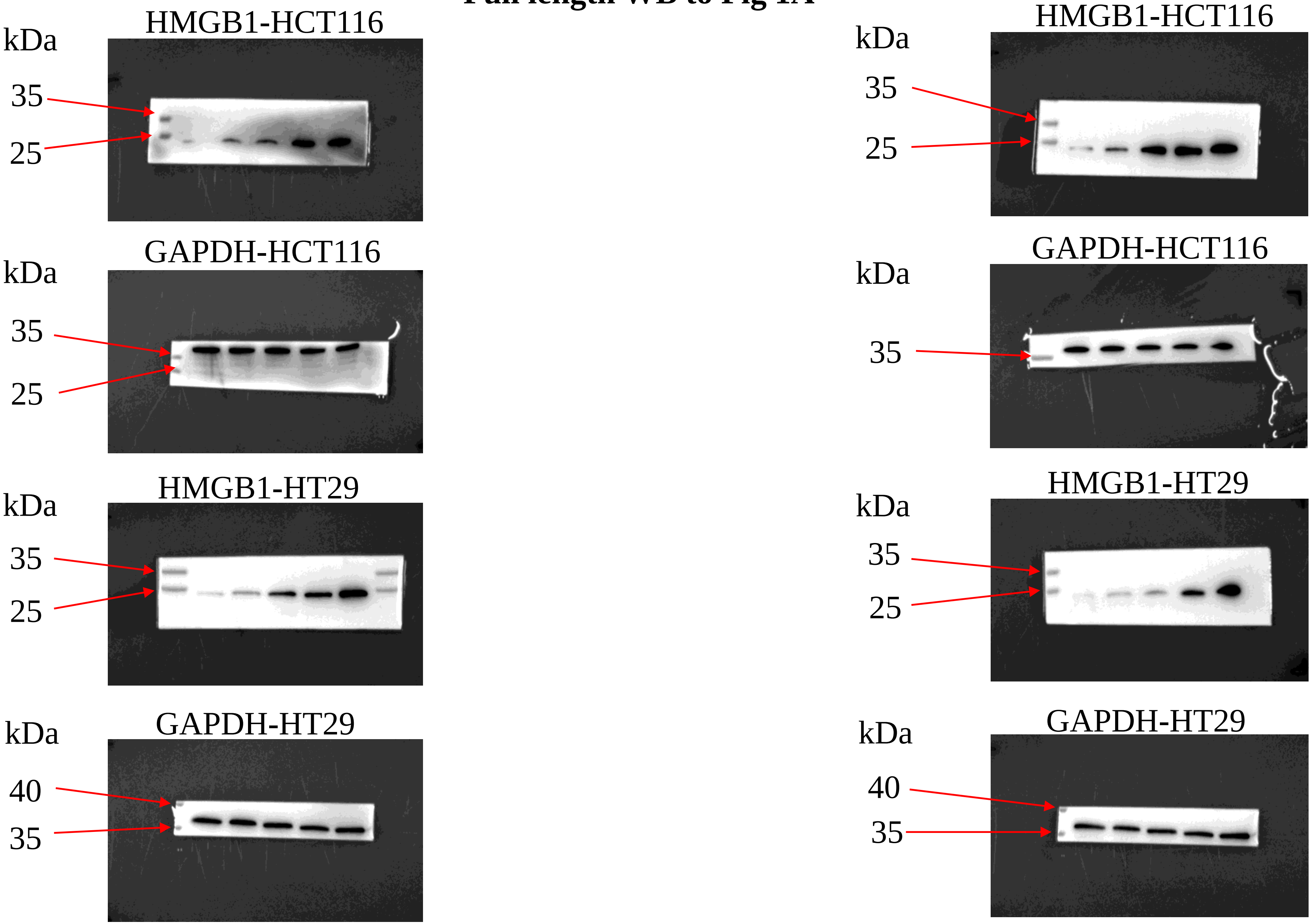

Full length WB to Fig 1B

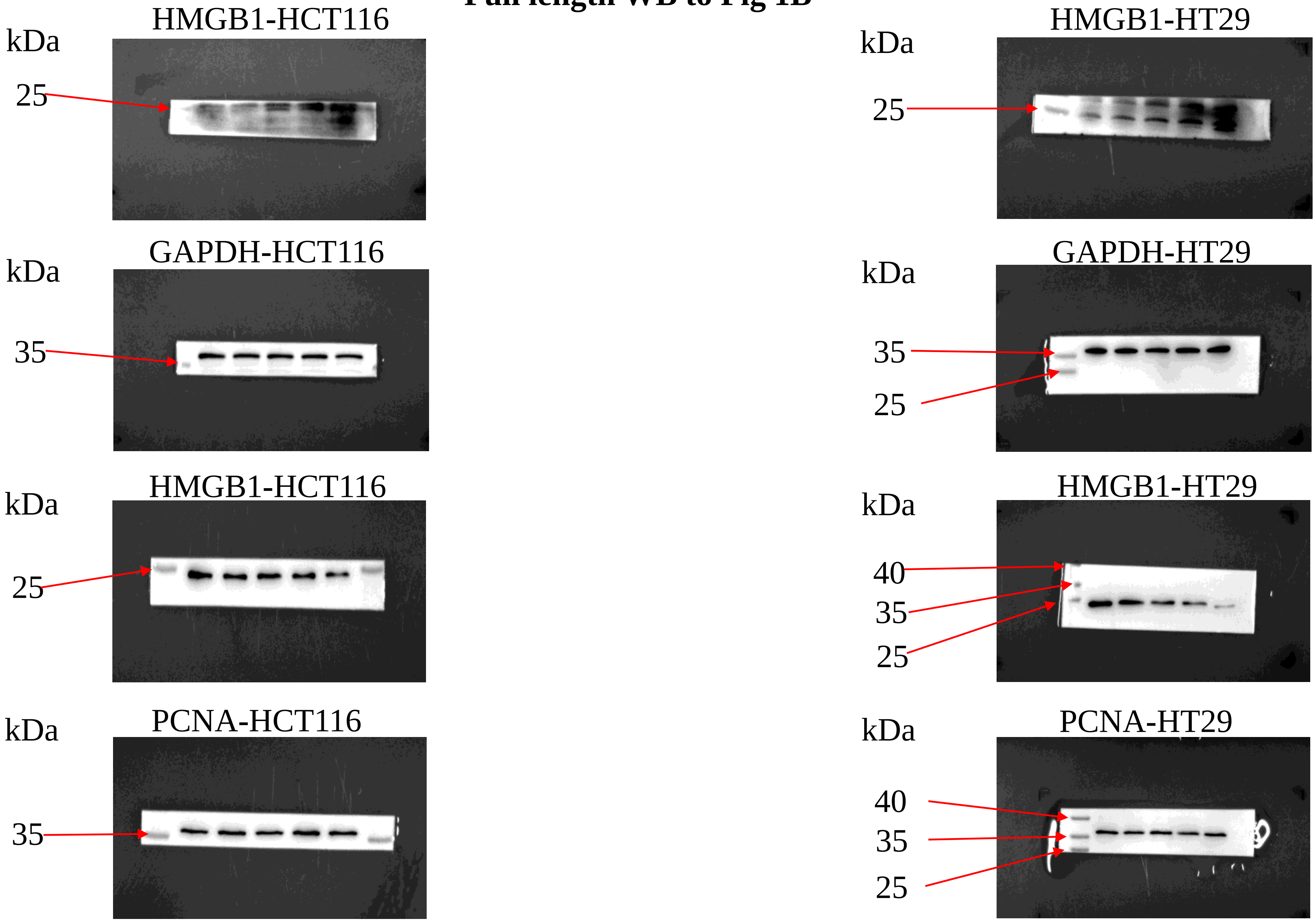

Full length WB to Fig 1C

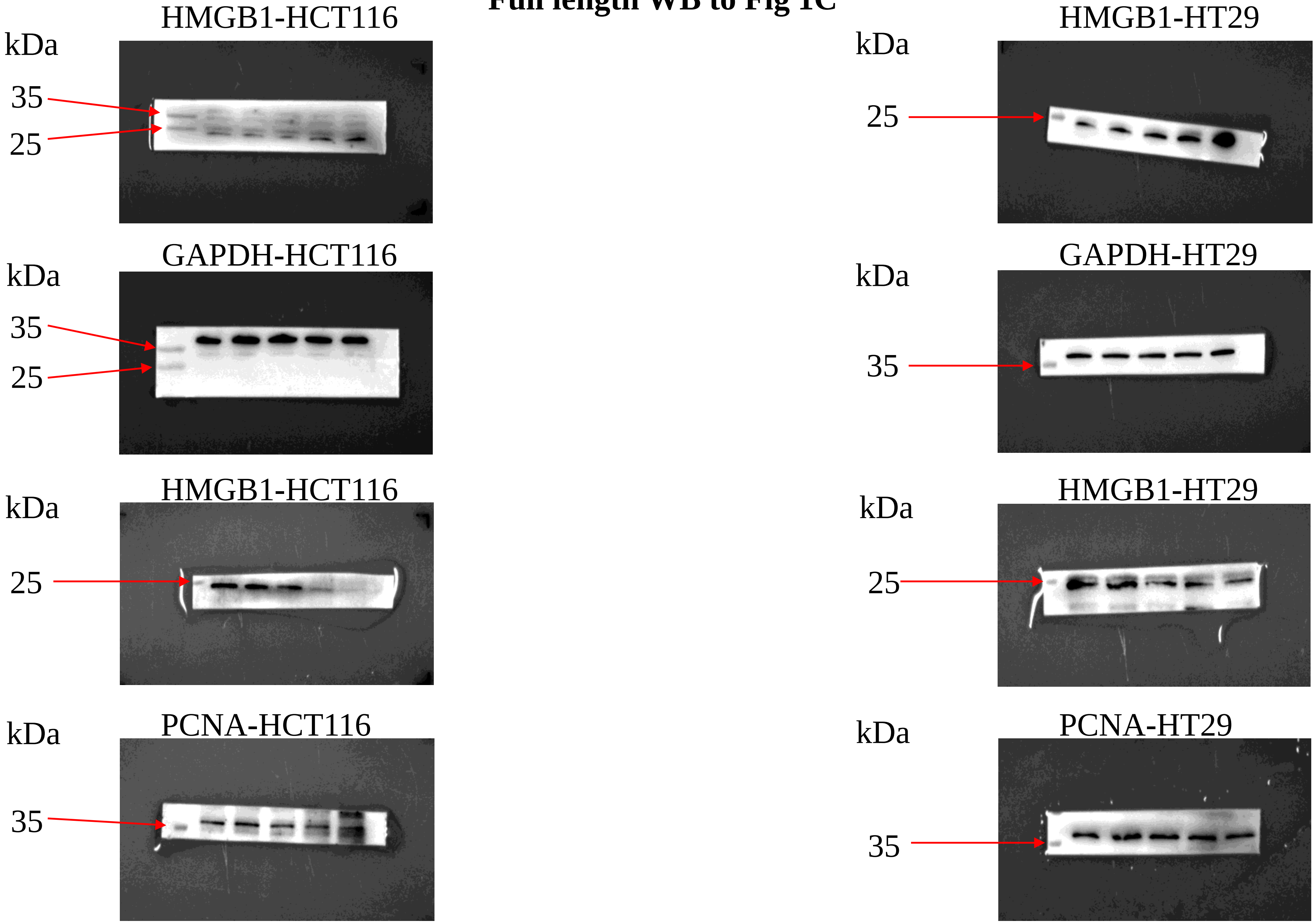

Full length WB to Fig 2

Fig 2B

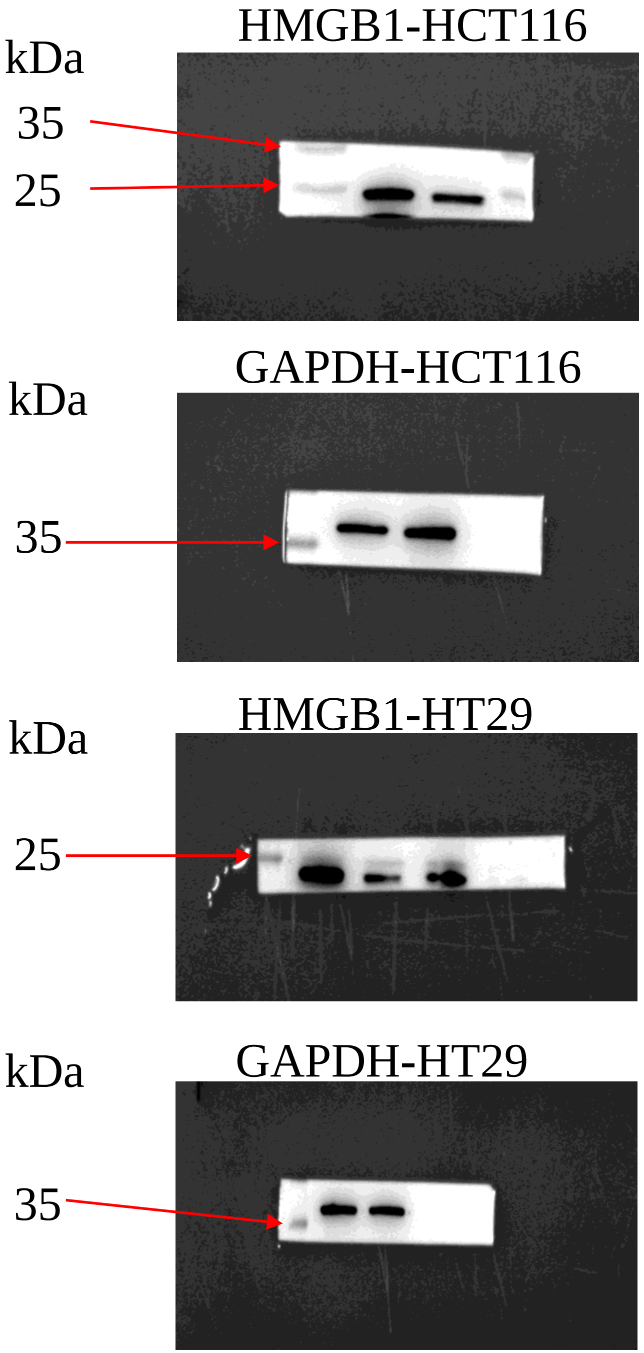

Fig 2F

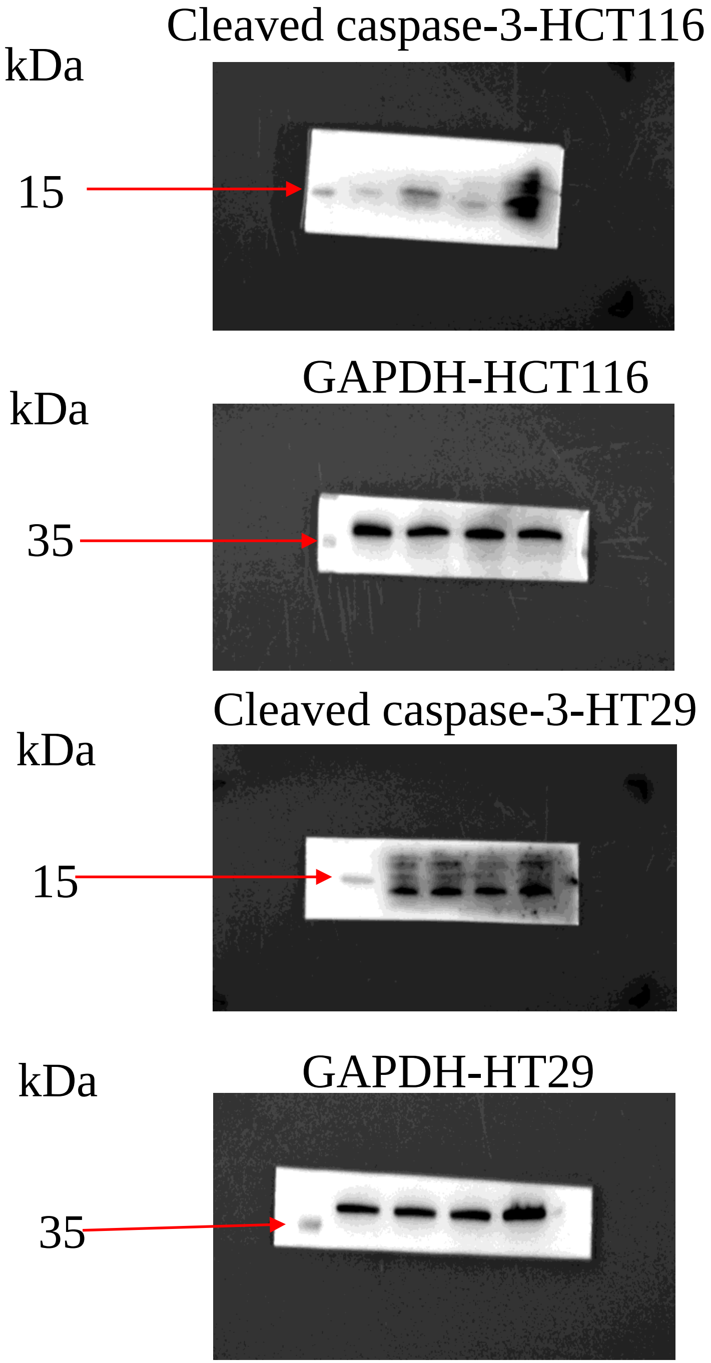

Full length WB to Fig 2H

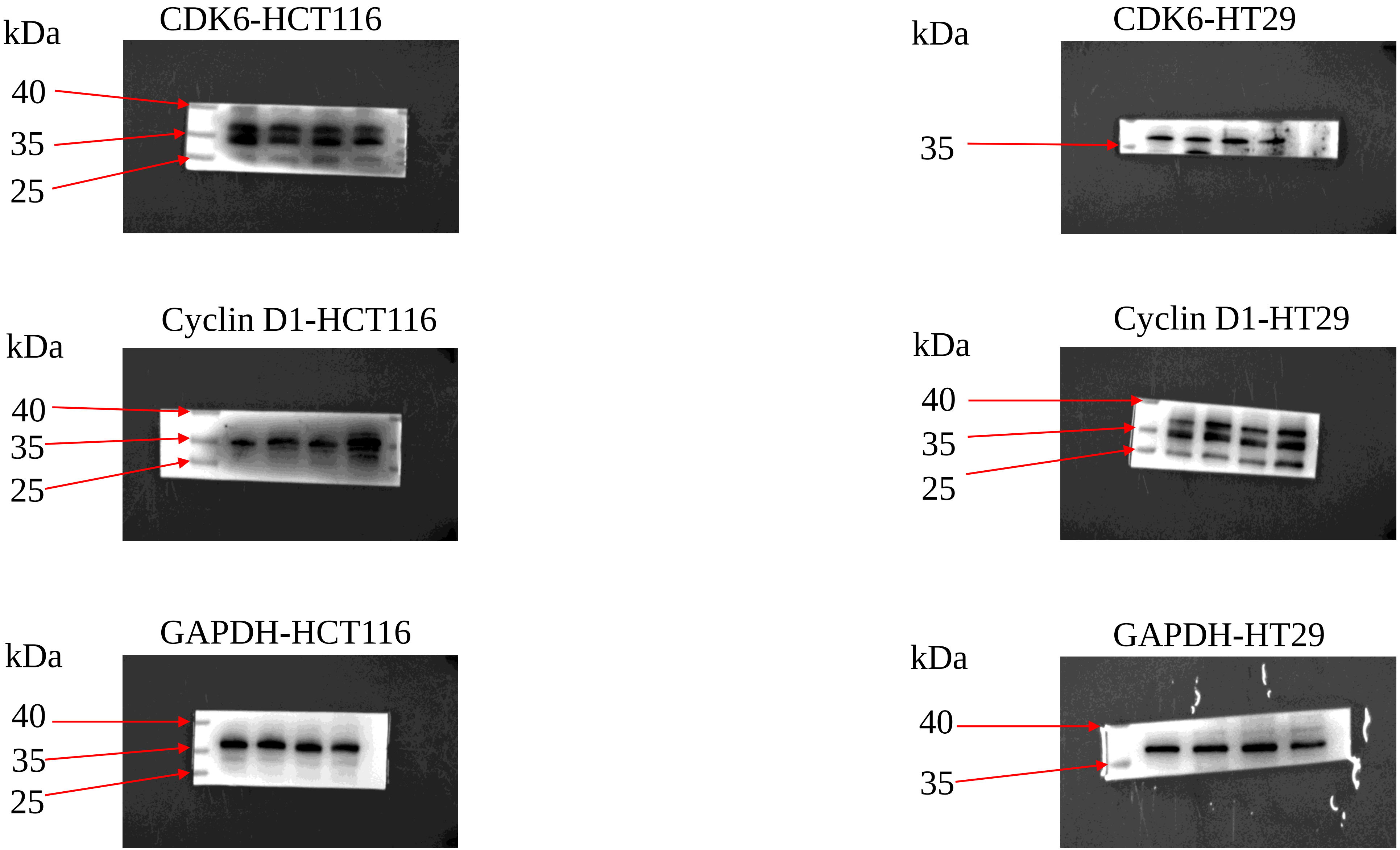

Full length WB to Fig 4A

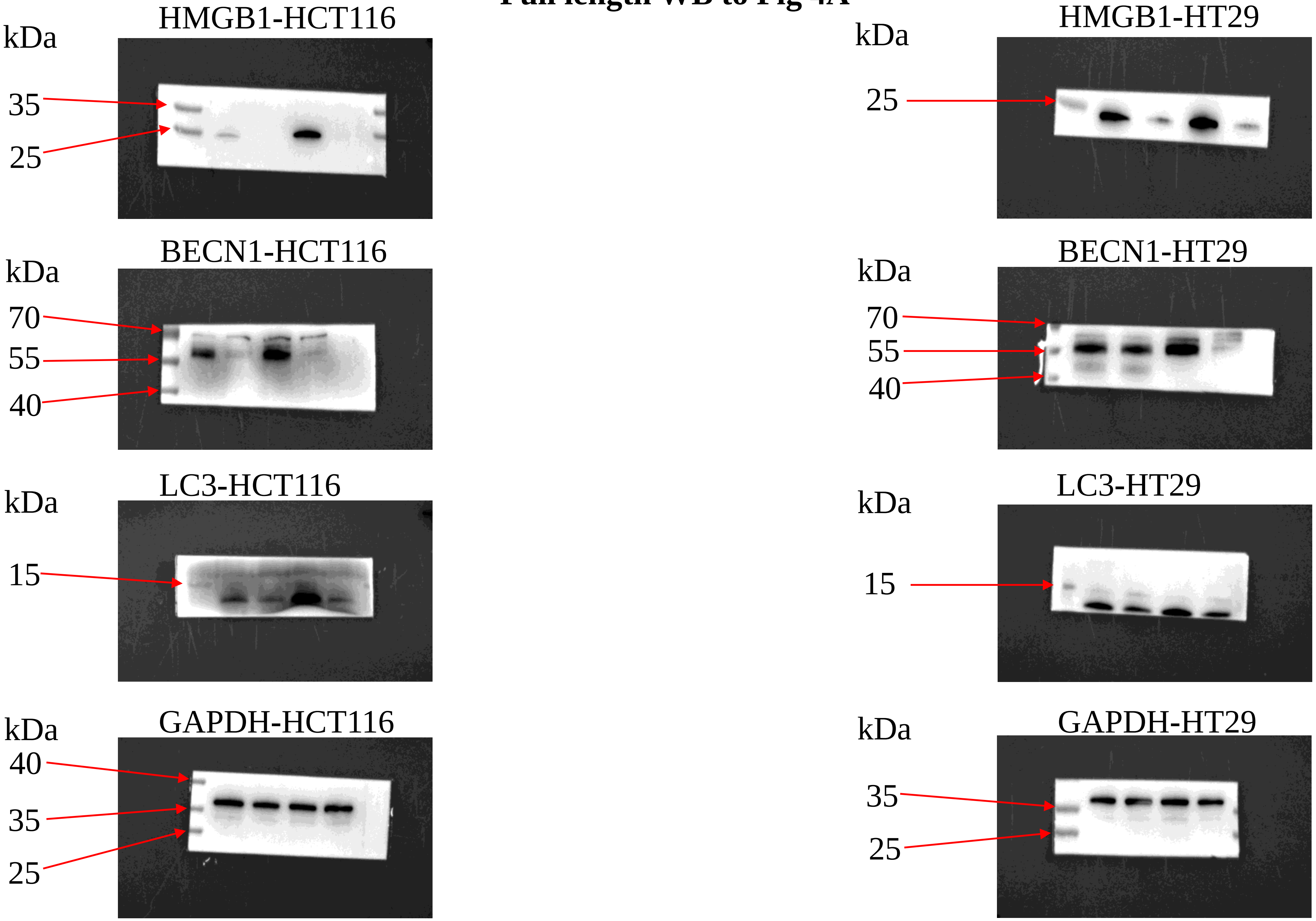

Full length WB to Fig 4B

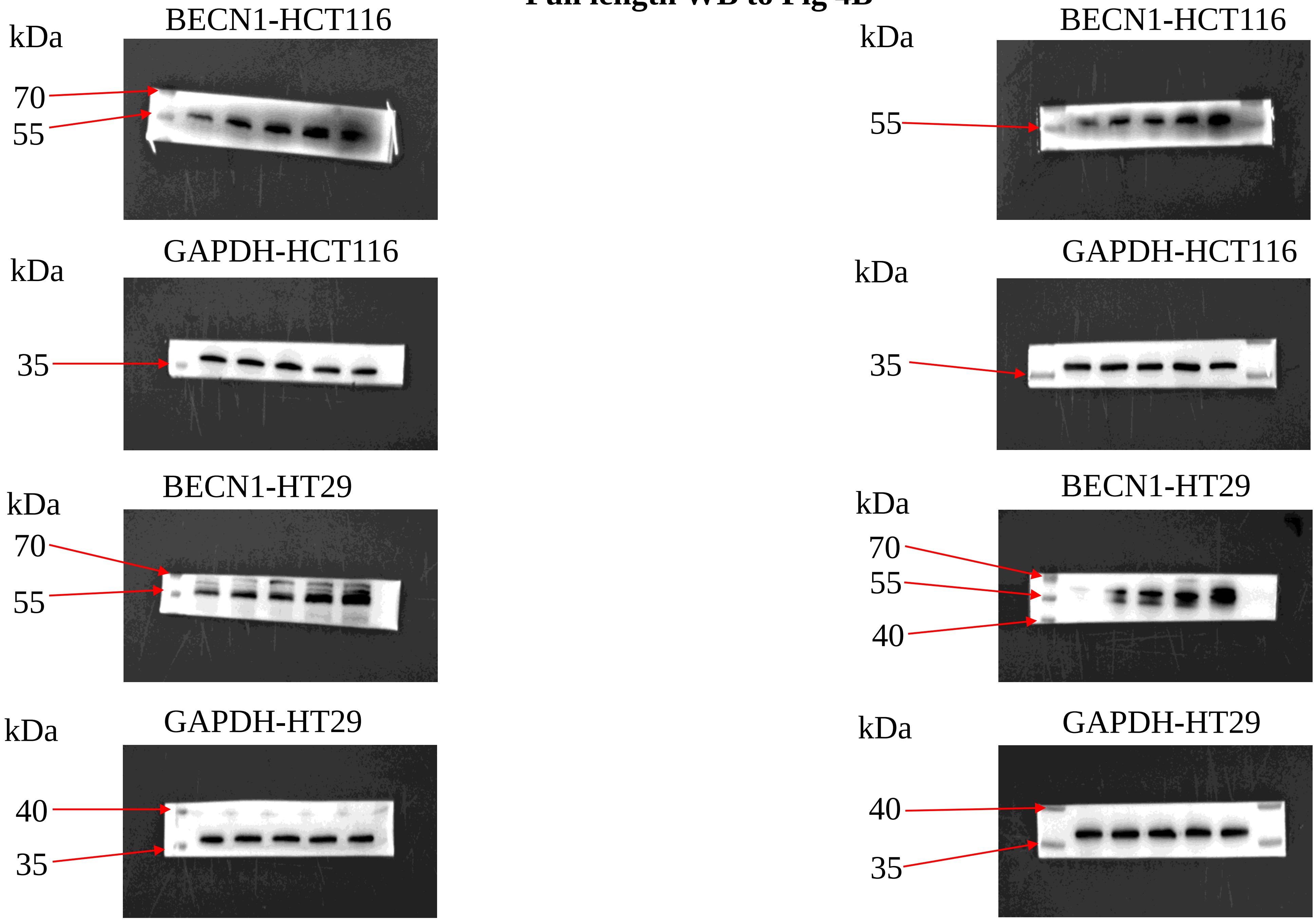

Full length WB to Fig 4

Fig 4C

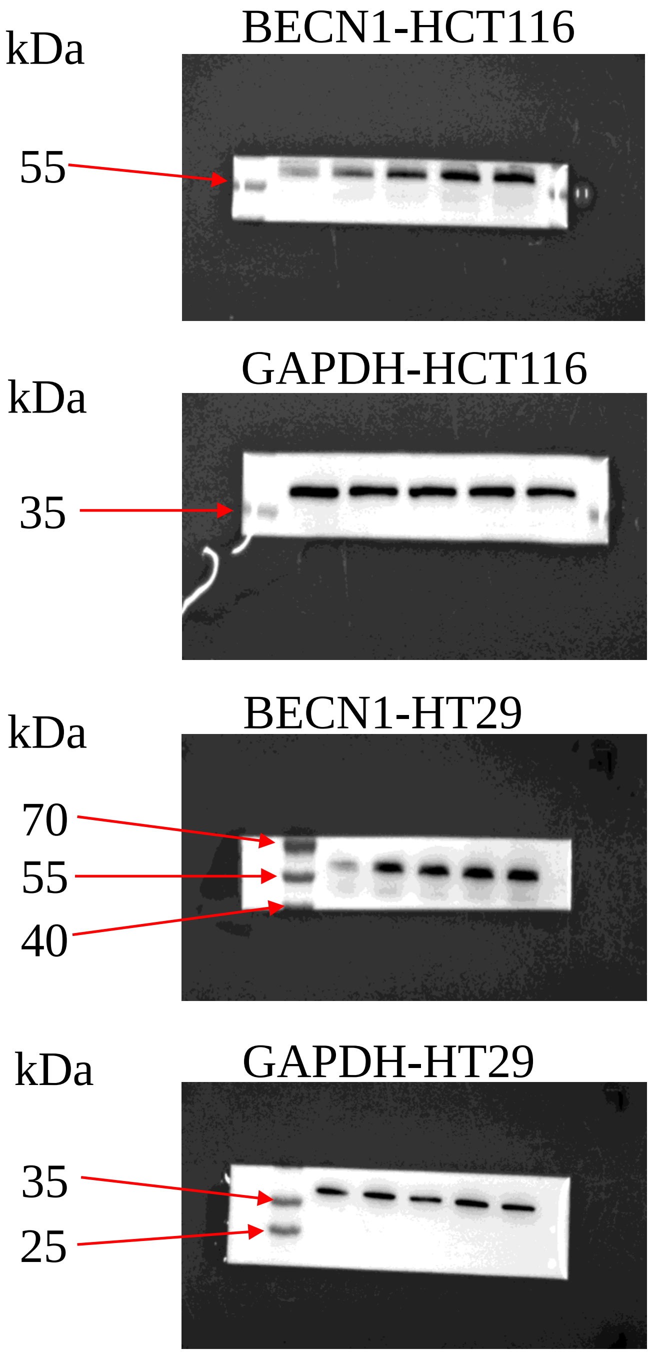

Fig 4D

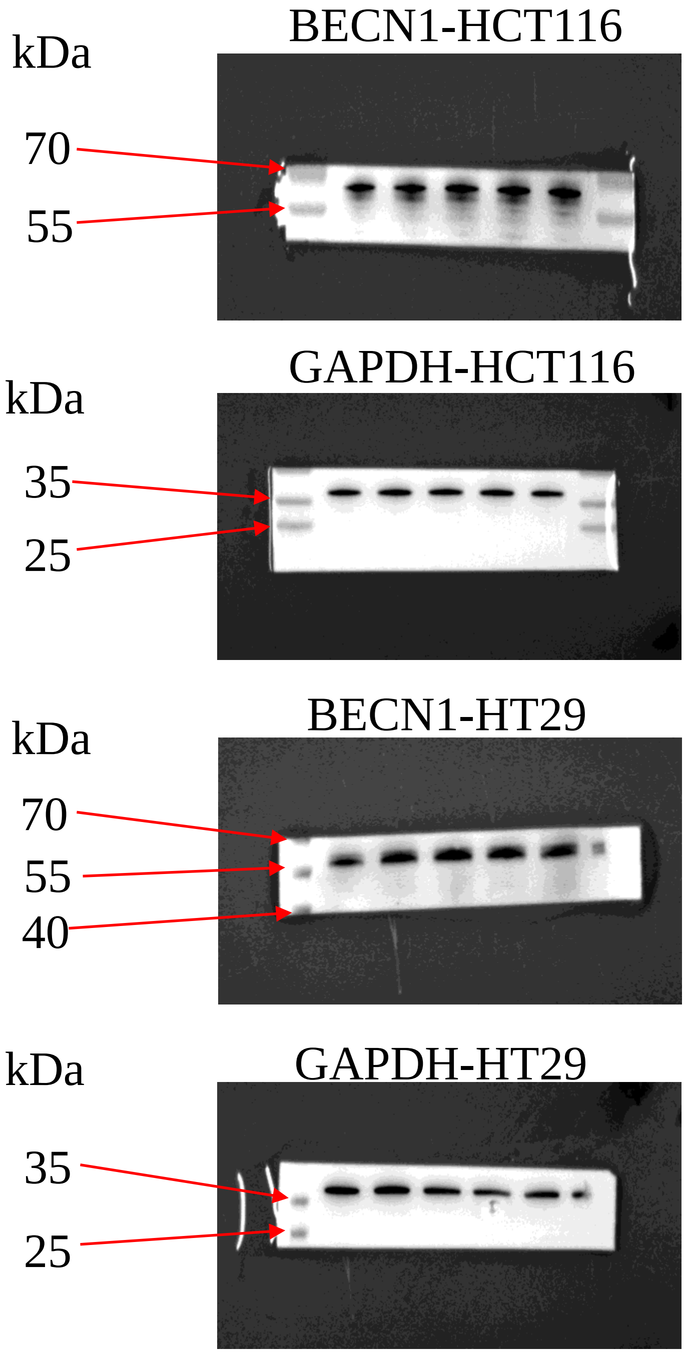

Full length WB to Fig 4E

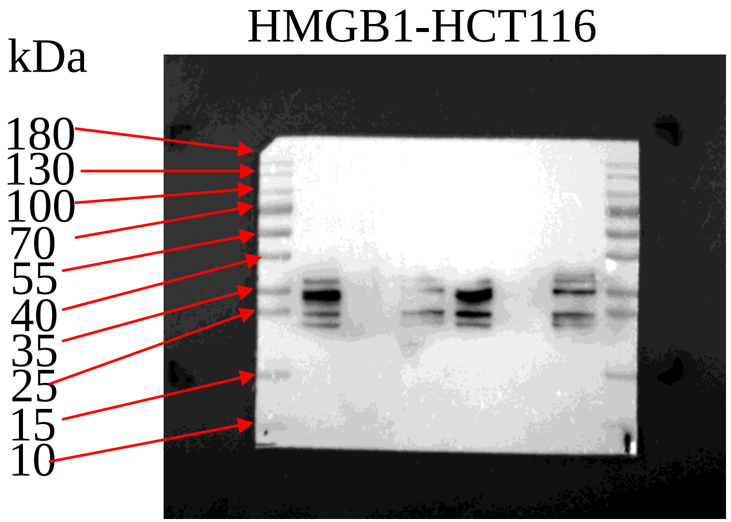

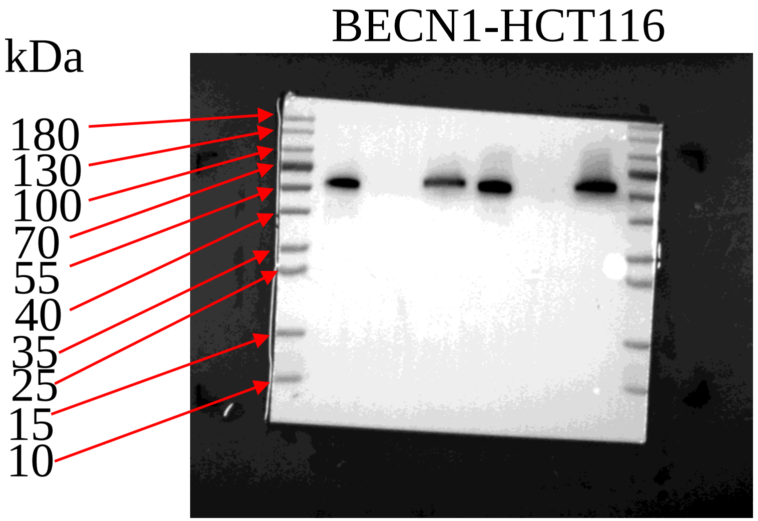

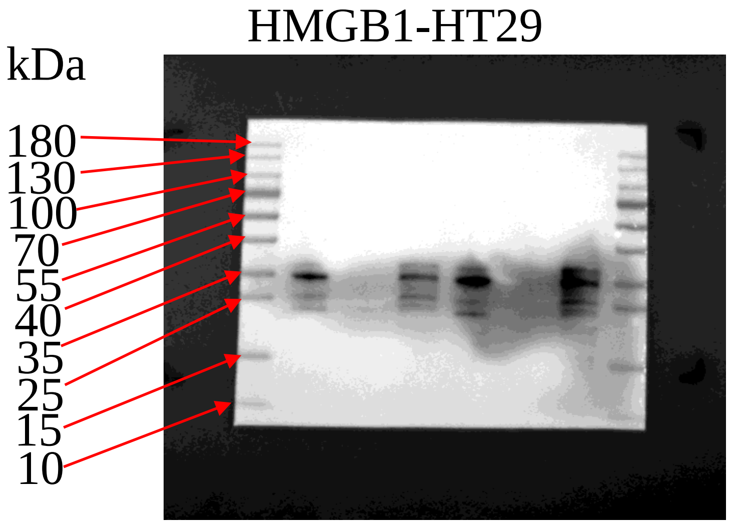

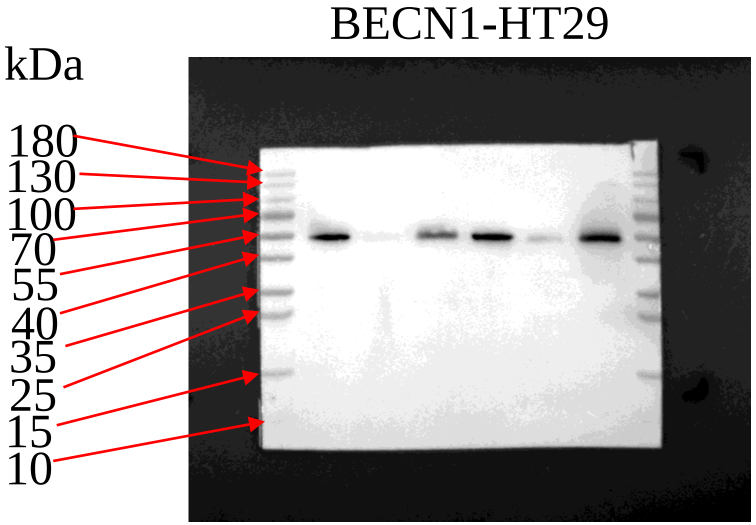

Full length WB to Fig 5

Fig 5D

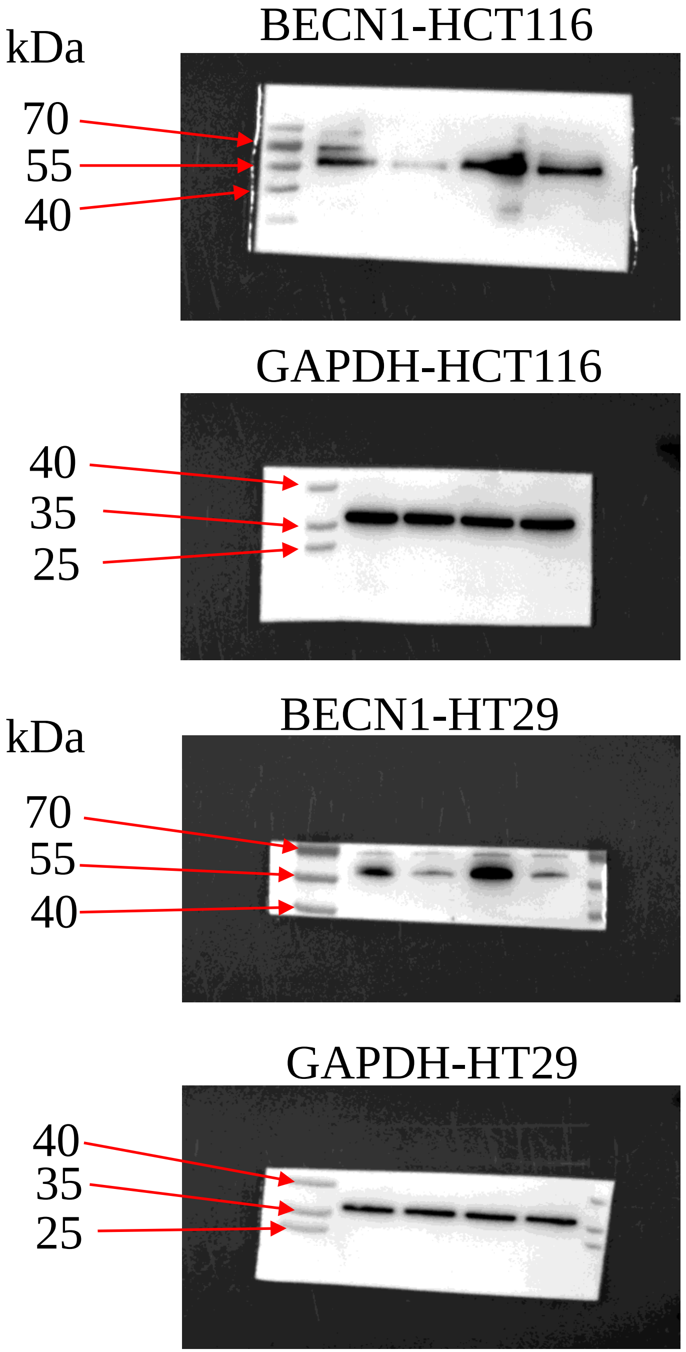

Fig 5G

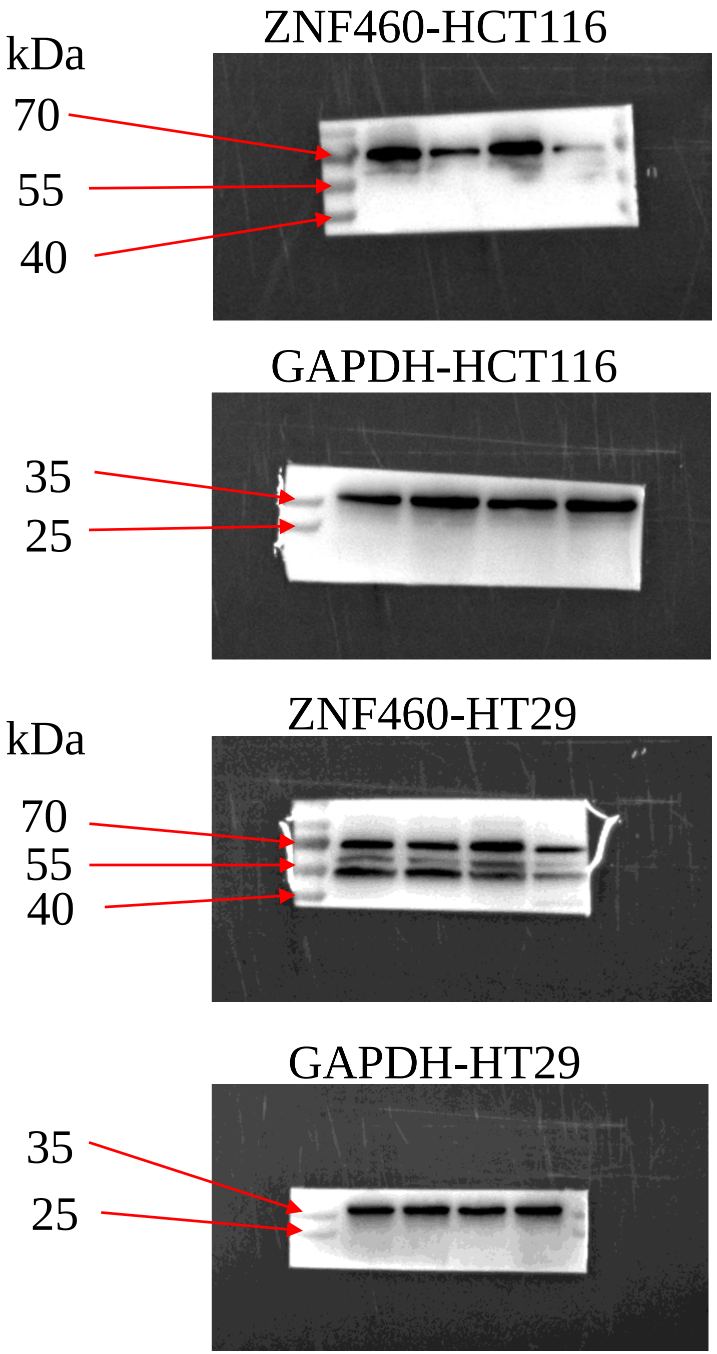

Full length WB to Fig 6E

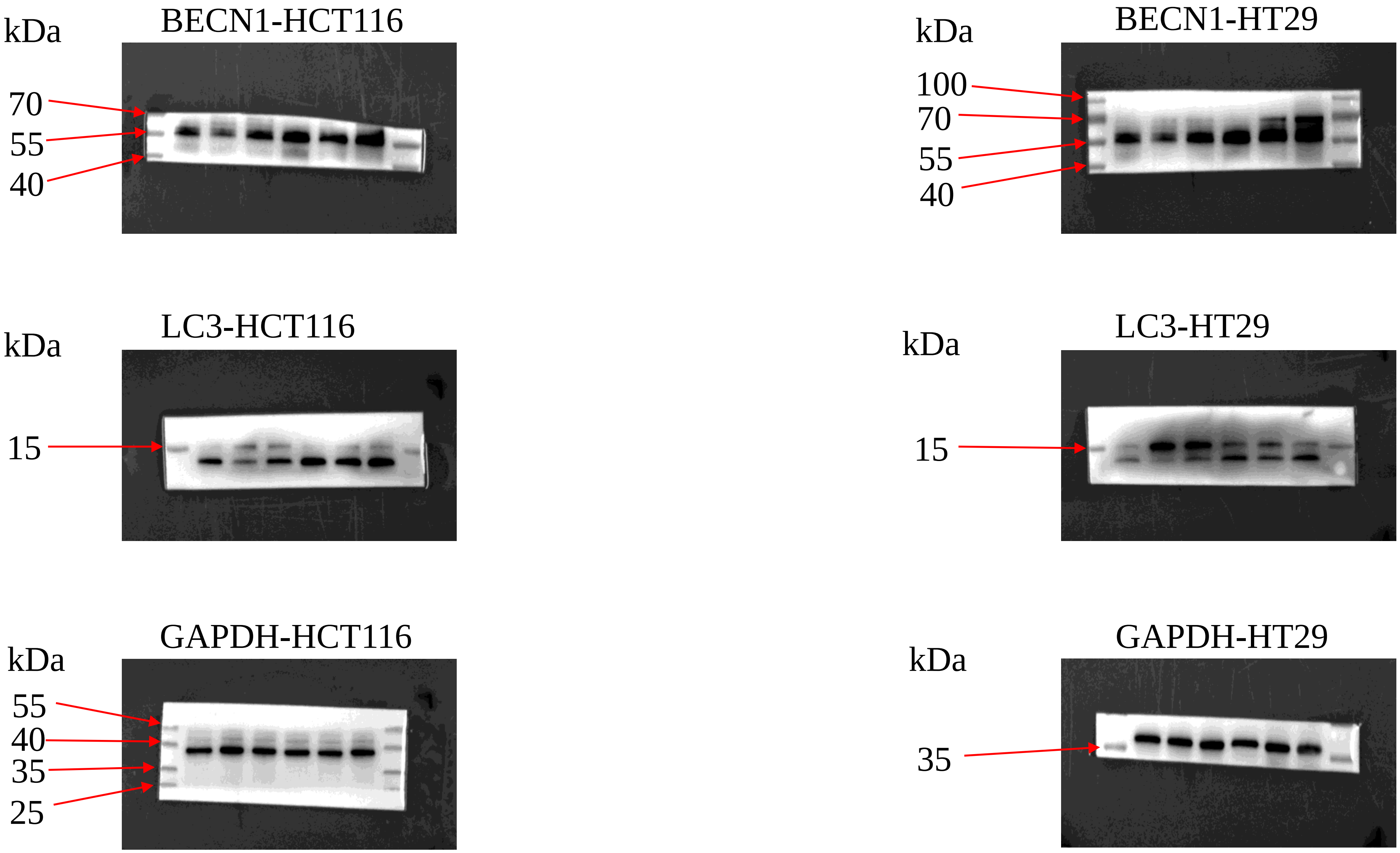

Full length WB to Fig s1

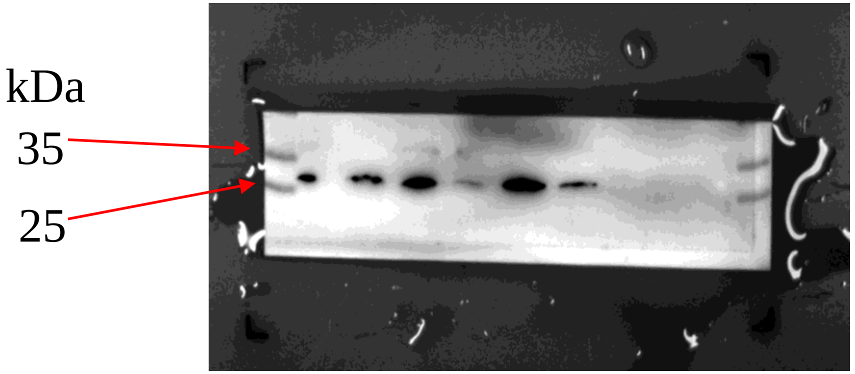

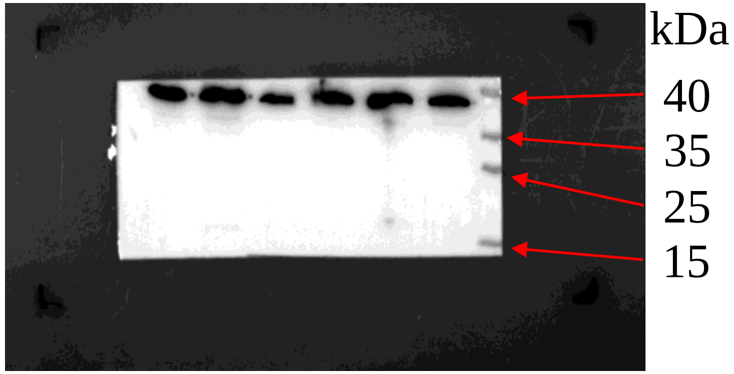

Full length WB to Fig S3D

kDa ZNF460-HCT116

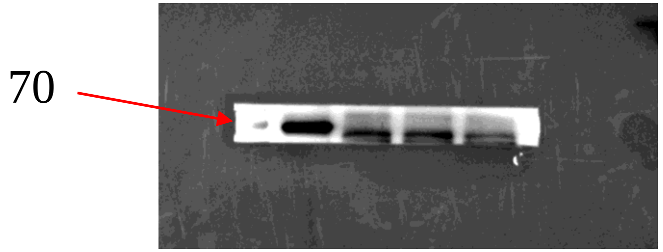

kDa ZNF460-HT29

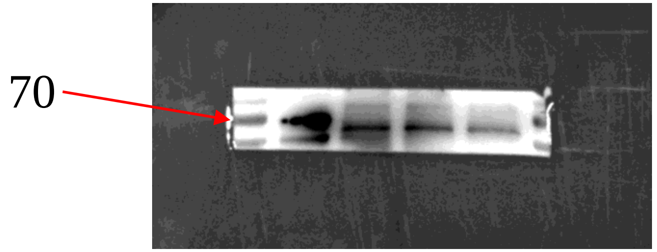

kDa GAPDH-HCT116

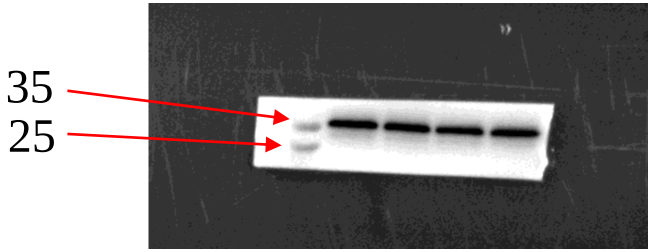

kDa GAPDH-HT29

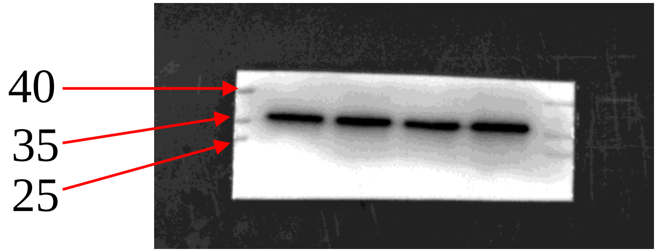

Full length WB to Fig S4

Fig s4B

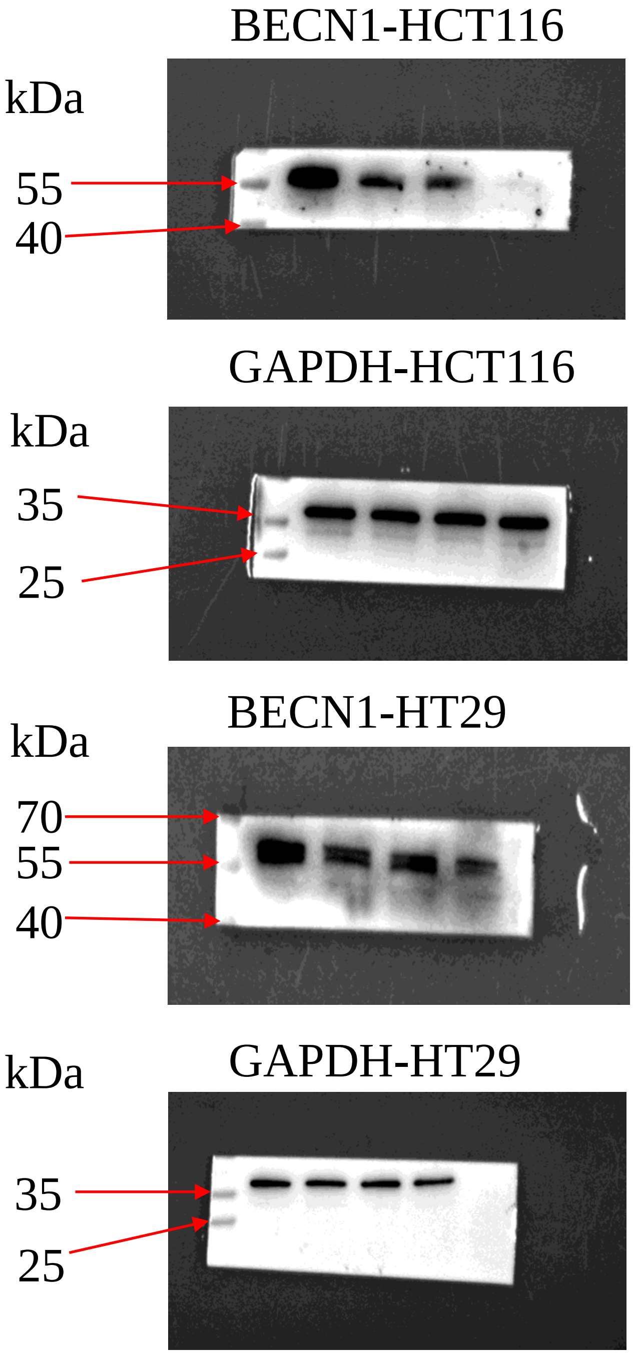

Fig s4E

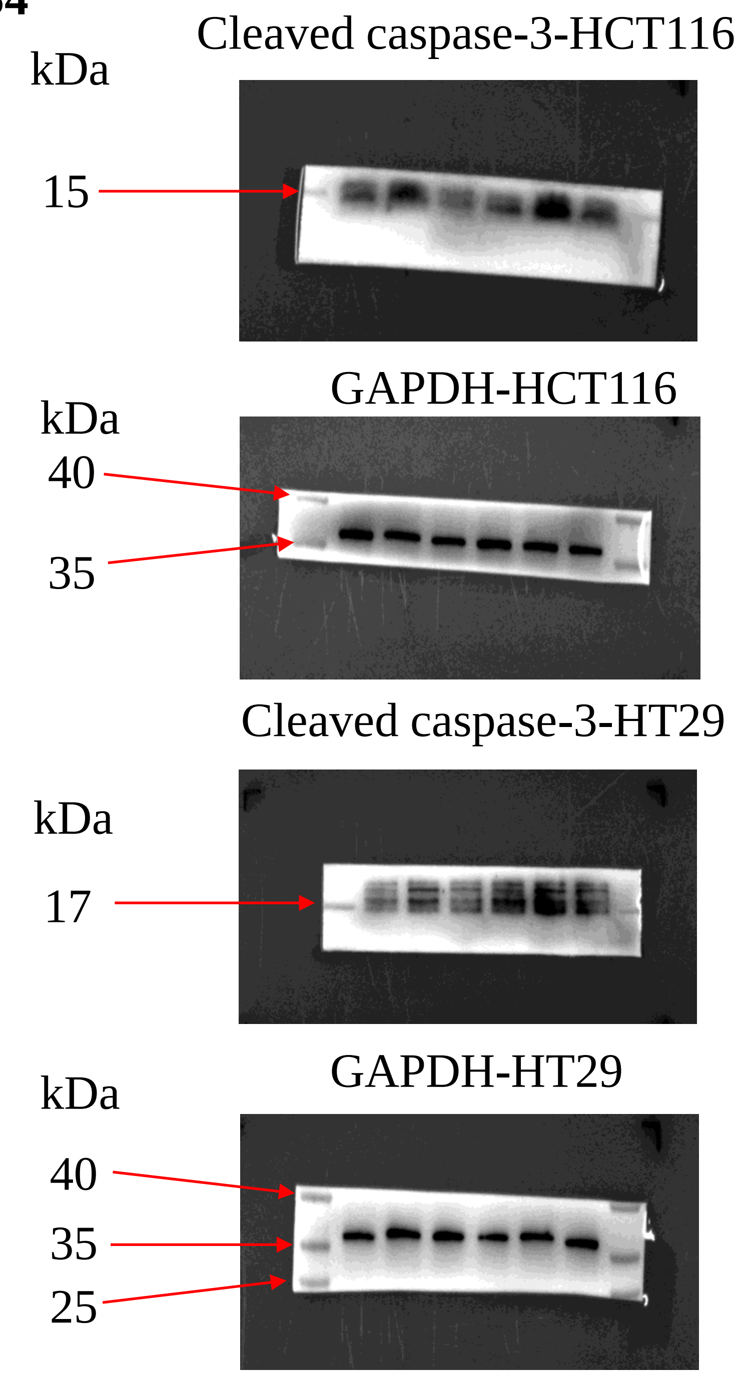

Full length WB to Fig s5B

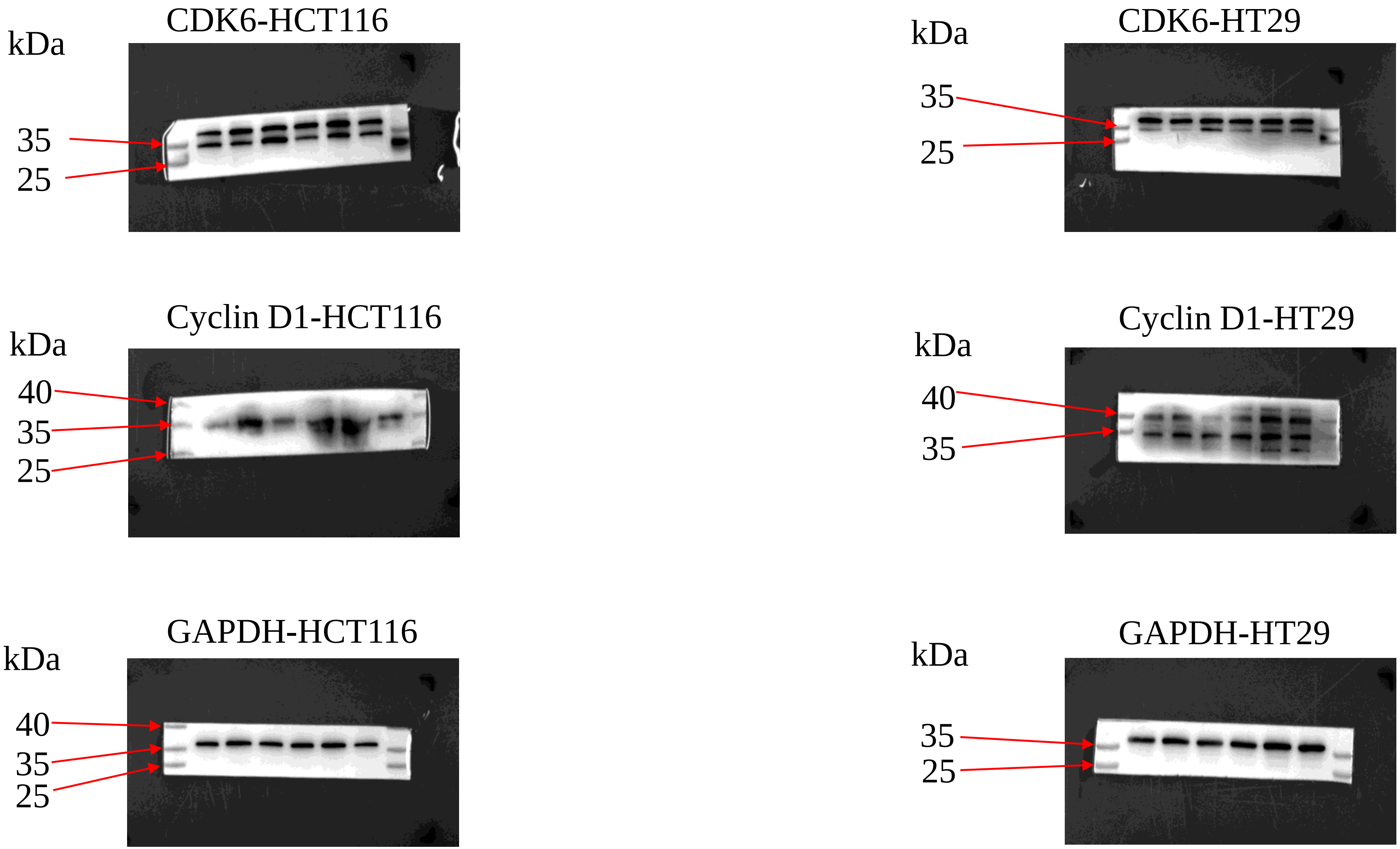

Supplement: Supplementary file 1 [file DataSheet1.pdf]
