# Supplementary material for: Cytoplasmic HMGB1 promotes and interacts with BECN1 through ZNF460 to induce autophagy and accelerate radioresistance in colorectal cancer cells
Source: Front Immunol. 2025 Oct 14;16:1642915. doi: 10.3389/fimmu.2025.1642915 (PMC12558843; doi:10.3389/fimmu.2025.1642915)
Supplement: Supplementary file 2 [file DataSheet2.docx]

**Supplementary materials for**

**Cytoplasmic HMGB1 Promotes Interaction with BECN1 via ZNF460 to Induce Autophagy and Enhance Radioresistance in Colorectal Cancer**

**Supplementary Materials and Methods**

**Table s1. Primers for the RT‒qPCR assay of genes.**

| Primer Name | Primer Sequence (5’-3’) |
| --- | --- |
| Homo-HMGB1 Forward | TATGGCAAAAGCGGACAAGG |
| Homo-HMGB1 Reverse | CTTCGCAACATCACCAATGGA |
| Homo-BECN1 Forward | CCATGCAGGTGAGCTTCGT |
| Homo-BECN1 Reverse | GAATCTGCGAGAGACACCATC |
| Homo-GAPDH Forward | GGAGCGAGATCCCTCCAAAAT |
| Homo-GAPDH Reverse | GGCTGTTGTCATACTTCTCATGG |
| Homo-ZNF460 Forward | CAAGGCACCAGCGGATTCAC |
| Homo-ZNF460 Reverse | CTCAGTGTGGATGATGGAGTGTTG |

**Table s2. Antibody information.**

| Antibody | Application | Cat. No | Dilution |
| --- | --- | --- | --- |
| HMGB1 | Western blotting | CST Cat# 6893s, RRID: AB_10827882 | 1:3000 |
| Cyclin D1 | Western blotting | Proteintech Cat# 60186-1-Ig, RRID: AB_10793718 | 1:20000 |
| CDK6 | Western blotting | Proteintech Cat# 14052-1-AP, RRID: AB_10642144 | 1:3000 |
| Cleaved caspase-3 | Western blotting | Proteintech Cat# 66470-2-Ig, RRID: AB_2876892 | 1:2000 |
| BECN1 | Western blotting | Proteintech Cat# 66665-1-Ig, RRID: AB_2882020 | 1:20000 |
| LC3 | Western blotting | Proteintech Cat# 14600-1-AP, RRID: AB_2137737 | 1:3000 |
| GAPDH | Western blotting | Proteintech Cat# 10494-1-AP, RRID: AB_2263076 | 1:20000 |
| PCNA | Western blotting | Proteintech Cat# 60097-1-Ig, RRID: AB_2236728 | 1:30000 |
| ZNF460 | Western blotting | ImmunoWay Cat# YT4968, RRID: AB_3675528 | 1:1000 |
| Ki67 | IHC | Proteintech Cat# 28074-1-AP, RRID: AB_2918145 | 1:1000 |
| HMGB1 | IHC | Proteintech Cat# 66525-1-Ig, RRID: AB_2881888 | 1:1000 |
| BECN1 | IHC | Proteintech Cat# 66665-1-Ig, RRID: AB_2882020 | 1:1000 |
| HMGB1 | Co-IP | Proteintech Cat# 66525-1-Ig, RRID: AB_2881888 | 5 μg |
| BECN1 | Co-IP | Proteintech Cat# 11306-1-AP, RRID: AB_2259061 | 5 μg |
| HMGB1 | PLA | ImmunoWay Cat# YT5502, RRID: AB_3675527 | 1:100 |
| BECN1 | PLA | Proteintech Cat# 66665-1-Ig, RRID: AB_2882020 | 1:100 |

**Table s3. Clinical characteristics of patients**

| CRC patients | Number | HMGB1 expression  *P* value | BECN1 expression  *P* value |
| --- | --- | --- | --- |
| No. of patients | 80 |  |  |
| Gender |  | 0.3618 | 0.7955 |
| Male | 58 |  | |
| Female | 22 |  |  |
| Age (years) |  | 0.9370 | 0.3972 |
| Mean | 56.88 |  | |
| Range | 33-76 |  |  |
| Pathology diagnosis | | | |
| Adenocarcinoma | 78 |  | |
| Mucinous adenocarcinoma | 2 |  |  |
| TNM stage |  |  |  |
| I-II | 44 | *P* < 0.01 | |
| III-IV | 36 |  |  |

**Figure Legends**

**Fig. s1. The expression of HMGB1 in CRC cells.** RT‒qPCR and western blotting were used to analyze the expression of HMGB1 in CRC cells (HCT116, HT29, DLD-1, SW480 and SW620) and normal colonic epithelial cells (NCM460). ***P* < 0.01, **P* < 0.05.

**Fig. s2. HMGB1 knockdown reduces the cell cycle distribution and promotes cell apoptosis. A.** Apoptosis was measured via Annexin V/7-AAD double staining in HMGB1-knockdown CRC cells after exposure to 4 Gy X-ray irradiation. **B.** The protein expression level of cleaved caspase 3 in HMGB1-knockdown CRC cells after exposure to 4 Gy X-ray irradiation was analyzed via ImageJ. GAPDH served as a loading control. **C.** Effects of HMGB1 knockdown on the cell cycle distribution of HMGB1-knockdown CRC cells after exposure to 4 Gy X-ray irradiation. **D.** The protein expression levels of CDK6 and cyclin D1 in HMGB1-knockdown CRC cells after exposure to 4 Gy X-ray irradiation were analyzed via ImageJ. GAPDH served as a loading control. The values are expressed as the means ± SDs. ***P* < 0.01, **P* < 0.05.

**Fig. s3. BECN1 was increased after irradiation in CRC cells. A.** The protein expression level of BECN1 after irradiation was analyzed via ImageJ. Left panel, the expression level of BECN1 after different doses of irradiation (0, 2, 4, 6, and 8 Gy). Right panel, the expression level of BECN1 at different time points (0, 3, 6, 12, and 24 h) after exposure to 4 Gy. **B.** The protein expression level of BECN1 in the cytoplasm of CRC cells after different doses of irradiation (0, 2, 4, 6, and 8 Gy) were analyzed via ImageJ. **C.** The protein expression of BECN1 in the cytoplasm of CRC cells at different time points (0, 3, 6, 12, and 24 h) exposed to 4 Gy was analyzed via ImageJ. **D.** Western blotting and RT‒qPCR were used to analyze the expression of ZNF460 in CRC cells after transfection with ZNF460 siRNA. The values are expressed as the means ± SDs. ***P* < 0.01, **P* < 0.05.

**Fig. s4. The HMGB1/BECN1 axis confers** **resistance to irradiation *in CRC cells in vitro*.** RT‒qPCR **(A)** and western blotting **(B)** were used to analyze the expression of BECN1 in CRC cells after transfection with BECN1 siRNA. **C.** The percentage of viable HCT116 and HT29 cells at 24 h post-Tat-BECN1 treatment, ranging from 0–10 µM, was assessed via CCK8 assays. **D.** Apoptosis was measured via Annexin V/7-AAD double staining in HMGB1-knockdown CRC cells treated with BECN1 siRNA or Tat-BECN1 prior to treatment with 4 Gy X-ray irradiation. **E.** The protein expression of cleaved caspase 3 in HMGB1-knockdown CRC cells treated with Tat prior to 4 Gy X-ray irradiation. GAPDH served as a loading control. ***P* < 0.01, **P* < 0.05.

**Fig. s5. The HMGB1/BECN1 axis confers resistance to irradiation *in CRC cells both in vitro* and *in vivo*. A.** Cell cycle progression was measured in HMGB1-knockdown CRC cells treated with BECN1 siRNA or Tat prior to 4 Gy X-ray irradiation. **B.** Protein expression of CDK6 and cyclin D1 in HMGB1-knockdown CRC cells treated with Tat prior to 4 Gy X-ray irradiation. GAPDH served as a loading control. The values are expressed as the means ± SDs. **C.** Protein expression of BECN1 and LC3 in HMGB1-knockdown CRC cells treated with Tat prior to 4 Gy X-ray irradiation. GAPDH served as a loading control. The values are expressed as the means ± SDs. **D.** Ki67 IHC staining of tumor tissues from the xenograft model with the indicated treatments (scale bar: 20 μm). **E.** TUNEL staining of tumor tissues from the xenograft model with the indicated treatments (scale bar: 20 μm). ***P* < 0.01, **P* < 0.05.
