# Supplementary figures and images for: Cytoplasmic HMGB1 promotes and interacts with BECN1 through ZNF460 to induce autophagy and accelerate radioresistance in colorectal cancer cells
Source: Front Immunol. 2025 Oct 14;16:1642915. doi: 10.3389/fimmu.2025.1642915 (PMC12558843; doi:10.3389/fimmu.2025.1642915)

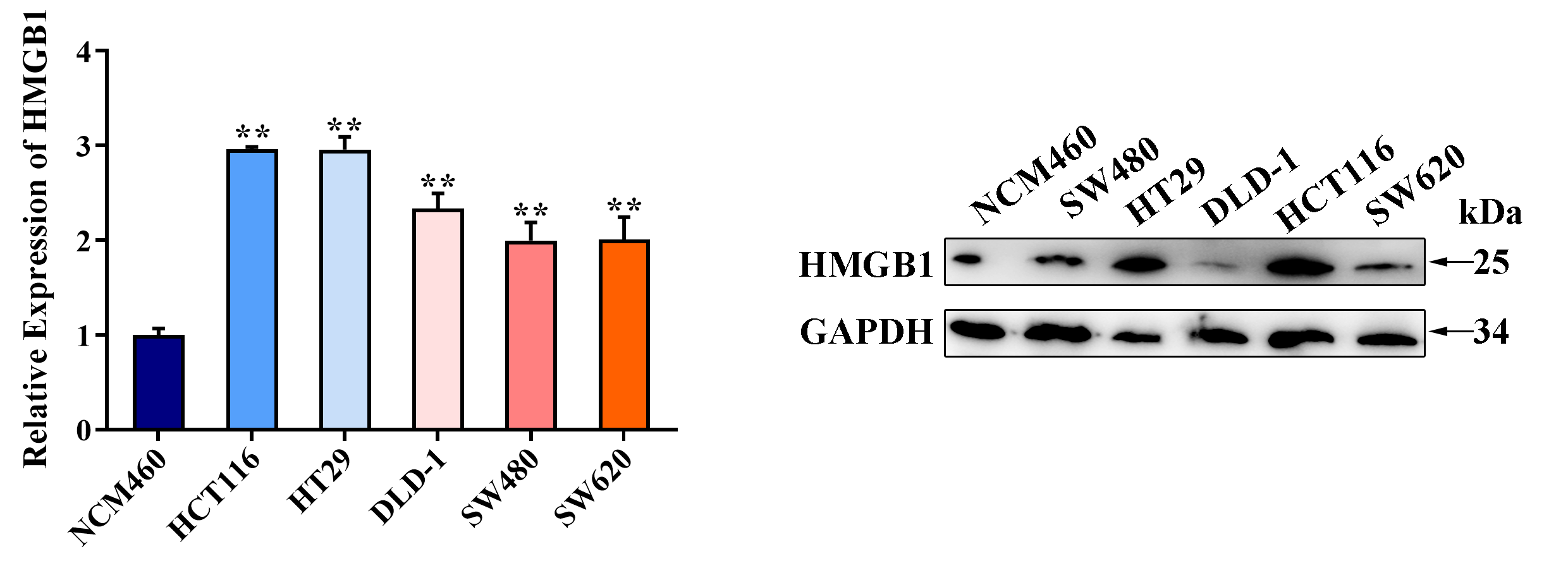

Supplement: Supplementary file 3 [file Image1.tif]

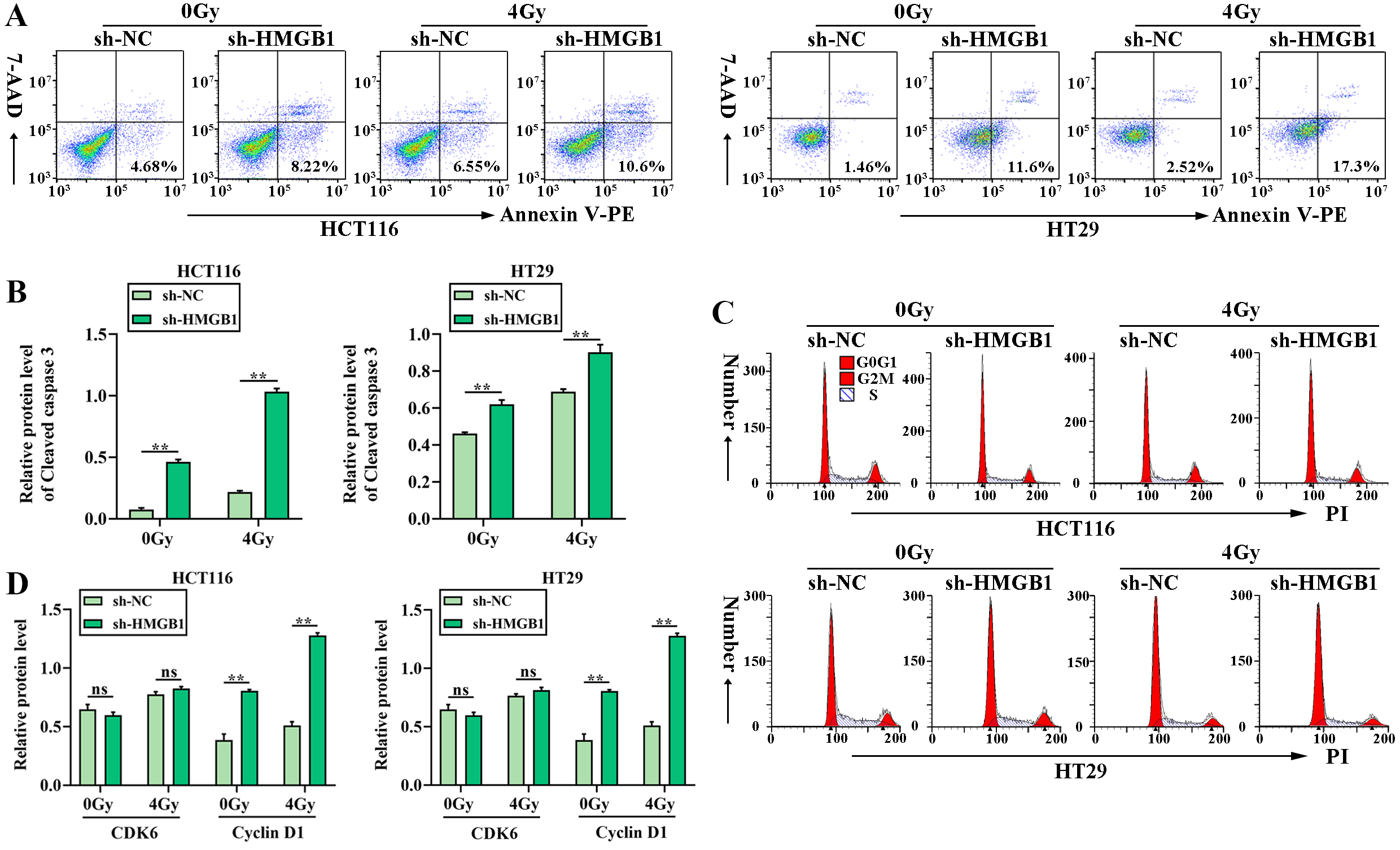

Supplement: Supplementary file 4 [file Image2.tif]

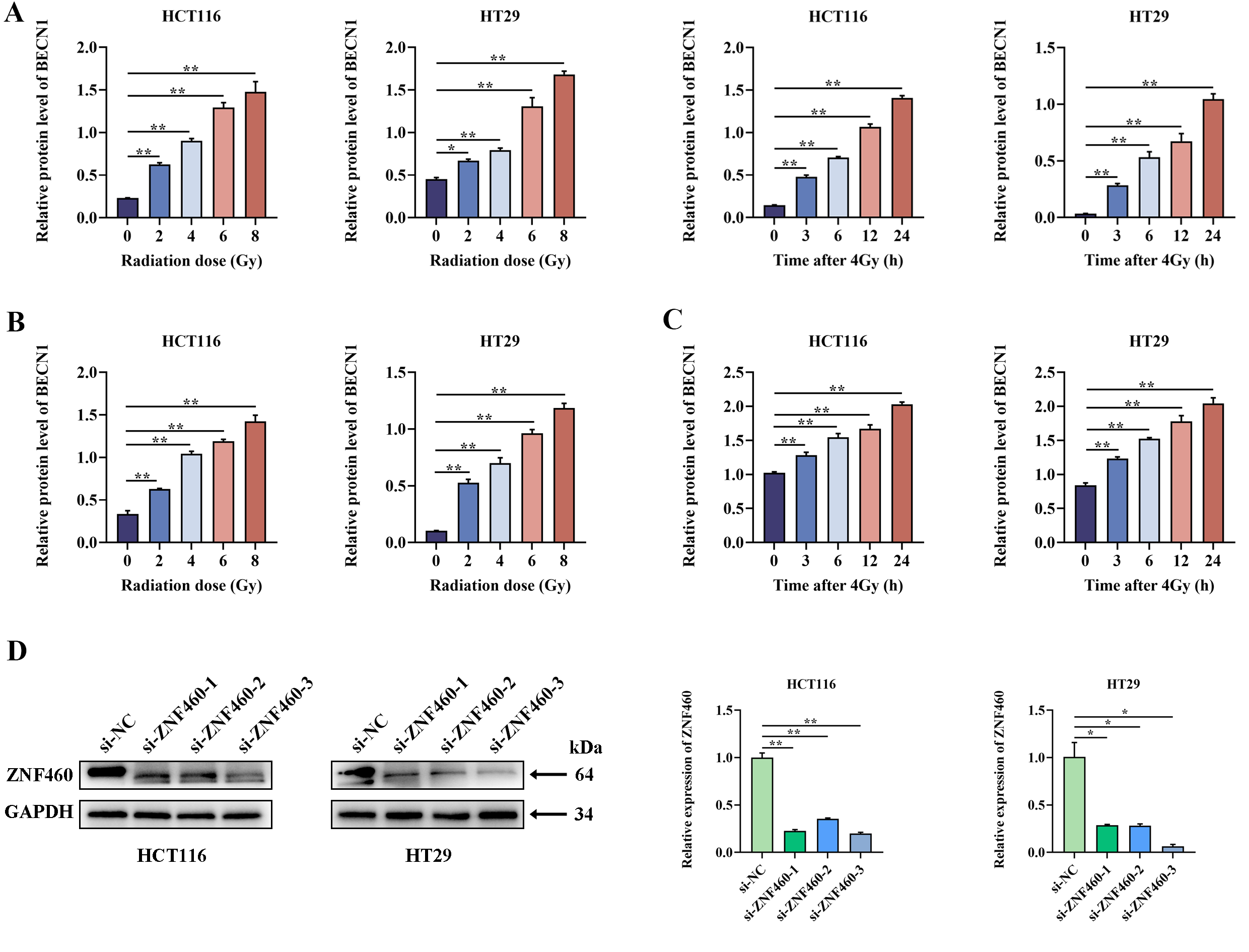

Supplement: Supplementary file 5 [file Image3.tif]

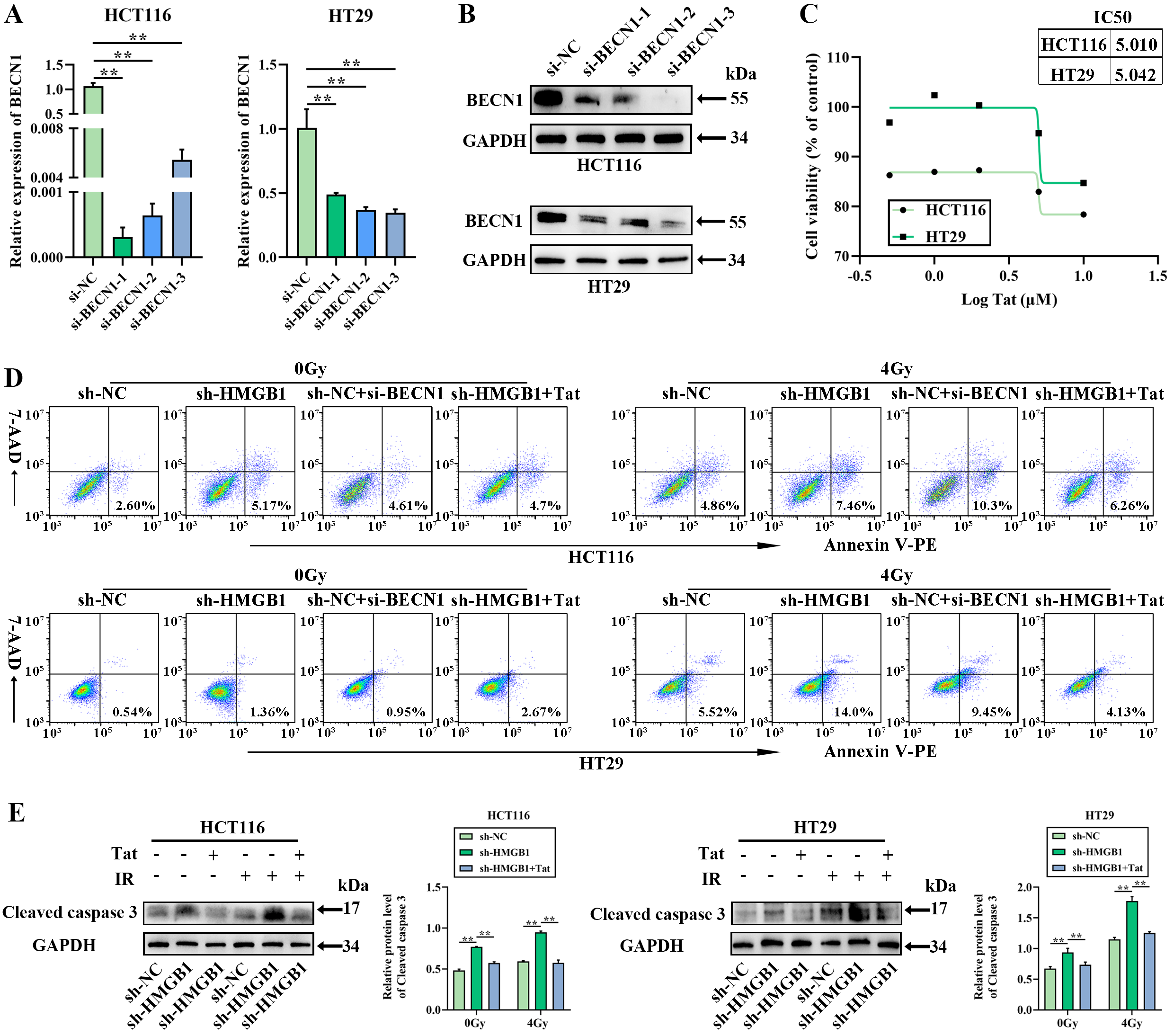

Supplement: Supplementary file 6 [file Image4.tif]

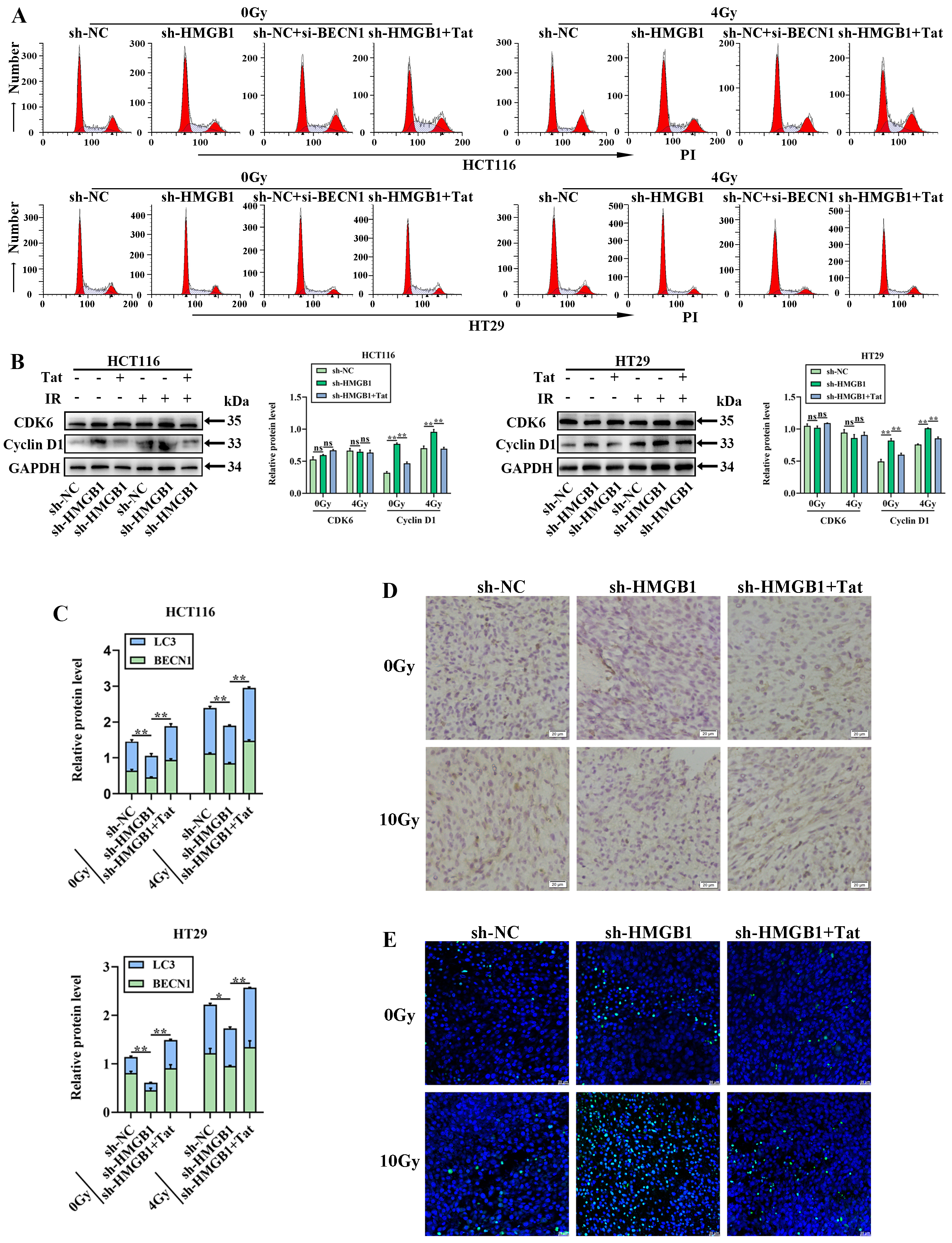

Supplement: Supplementary file 7 [file Image5.tif]
